# Adenosine A_2A_ receptor and ERK-driven impulsivity potentiates hippocampal neuroblast proliferation

**DOI:** 10.1038/tp.2017.64

**Published:** 2017-04-18

**Authors:** A Oliveros, C H Cho, A Cui, S Choi, D Lindberg, D Hinton, M-H Jang, D-S Choi

**Affiliations:** 1 Department of Molecular Pharmacology and Experimental Therapeutics, Mayo Clinic College of Medicine, Rochester, MN, USA; 2 Department of Neurologic Surgery, Mayo Clinic College of Medicine, Rochester, MN, USA; 3 Neurobiology of Disease Program, Mayo Clinic College of Medicine, Rochester, MN, USA; 4 Department of Biochemistry and Molecular Biology, Mayo Clinic College of Medicine, Rochester, MN, USA; 5 Department of Psychiatry and Psychology, Mayo Clinic College of Medicine, Rochester, MN, USA

## Abstract

Dampened adenosine A_2A_ receptor (A_2A_R) function has been implicated in addiction through enhancement of goal-directed behaviors. However, the contribution of the A_2A_R to the control of impulsive reward seeking remains unknown. Using mice that were exposed to differential reward of low rate (DRL) schedules during Pavlovian-conditioning, second-order schedule discrimination, and the 5-choice serial reaction time task (5-CSRTT), we demonstrate that deficits of A_2A_R function promote impulsive responses. Antagonism of the A_2A_R lowered ERK1 and ERK2 phosphorylation in the dorsal hippocampus (dHip) and potentiated impulsivity during Pavlovian-conditioning and the 5-CSRTT. Remarkably, inhibition of ERK1 and ERK2 phosphorylation by U0126 in the dHip prior to Pavlovian-conditioning exacerbated impulsive reward seeking. Moreover, we found decreased A_2A_R expression, and reduced ERK1 and ERK2 phosphorylation in the dHip of equilibrative nucleoside transporter type 1 (ENT1^–/–^) null mice, which displayed exacerbated impulsivity. To determine whether impulsive response behavior is associated with hippocampal neuroblast development, we investigated expression of BrdU^+^ and doublecortin (DCX^+^) following 5-CSRTT testing. These studies revealed that impulsive behavior driven by inhibition of the A_2A_R is accompanied by increased neuroblast proliferation in the hippocampus.

## Introduction

The dorsal hippocampus (dHip) and nucleus accumbens (NAc) are constituents of a neural circuit that regulates adaptability and survival.^1^ However, maladaptation of hippocampal-striatal circuits contribute to the development of impulsivity, compulsivity and addiction.^2^ Differential reinforcement of low rates of behavior (DRL) schedules have revealed the hippocampus to be a regulatory neural substrate in impulsivity and reward acquisition^3, 4^ in both human and animal models.^5, 6^ Alcohol-induced impulsivity in humans^7^ and rodents^8^ are partially driven by contextual evocation of drug-induced euphoria to cues. These contextual associations are largely regulated by the dHip, which has an instrumental role in controlling cue-induced drug seeking and relapse in substance use disorders.^9, 10^

As an inhibitory neurotransmitter, adenosine has an essential role in fine-tuning hippocampal-striatal circuits that drive learning, memory, motivation and addictive behaviors.^11, 12, 13^ The role of the adenosine A_2A_ receptor (A_2A_R) in brain physiology and behavior has recently gained significant attention due to its involvement in synaptic plasticity and memory.^14, 15, 16, 17^ Previously, we demonstrated that deletion of the ethanol-sensitive adenosine transporter (ENT1^–/–^) in mice decreases A_2A_R function in the dorsomedial striatum, resulting in increased ethanol consumption and enhanced goal-directed behavior.^18, 19^ ENT1 has been reported to regulate neurotransmission in the hippocampus.^20^ Hippocampal neurotransmission critically relies on activity of extracellular signal-regulated kinases (ERK1 and ERK2, also known as mitogen-activated protein kinase 3 (MAPK3) and MAPK1, respectively), and known to be essential components of synaptic plasticity and neurogenesis.^21, 22, 23^ Consequently, addictive drugs dampen hippocampal neurogenesis, which in turn can profoundly affect craving, relapse and cognition.^24, 25^

In this report, we utilized DRL-mediated Pavlovian-conditioning (DRL-conditioning), operant second-order discrimination, and the 5-choice serial reaction time task (5-CSRTT) to examine goal-and-sign-tracking impulsive behavior.^26^ Our results reveal the involvement of the A_2A_R and the downstream kinases ERK1/2 in impulsive reward seeking. Furthermore, we investigated a possible link between dysregulation of the A_2A_R in relation to neuroblast development within the hippocampal dentate gyrus (DG). Therefore, to our knowledge, our findings are the first to demonstrate a novel role for the A_2A_R in impulsivity and immature neuroblast proliferation, both of which may be factors underlying the process of neurogenesis in reward seeking behaviors.

## Materials and methods

Detailed materials and methods, including a list of behavioral groups and follow-up biological assessments are provided in the Supplementary Information and Supplementary Figure 1.

### Subjects

Age-matched 3-to-5 month ENT1^–/–^ and wild-type (WT) mice were generated in house, whereas age-matched 3-to-5 month C57BL/6J mice were ordered from Jackson Laboratories and cared for as previously described.^18, 19^ All experimental procedures were approved by the Mayo Clinic Institutional Animal Care and Use Committee and performed in accordance with NIH guidelines.

#### Behavior naive mice

ENT1^–/–^, WT and C57BL/6J mice that were naive to behavior testing and were acutely administered ethanol, or were treated with ZM-241385 (5-days), were group housed with littermates and maintained in ventilated racks with ad libitum food and water until ready for brain extraction for subsequent western blot and immunofluorescence analysis (Supplementary Figure 1).

#### Behavior-tested mice

Behavior-tested groups of WT and ENT1^–/–^ mice, and vehicle and ZM-241385-treated C57BL/6J mice were either group housed or individually housed, and underwent food restriction to reach ~85% of their ad libitum feeding target weight (Supplementary Figure 1). Detailed methods are available in the Supplementary Information.

### Drugs

For all experiments utilizing the specific A_2A_R antagonist ZM-241385 (Tocris Bioscience, Bristol, UK), we administered (i.p.) the drug at a dose of 20 mg kg^−1^. The specific MEK inhibitor U0126 (Sigma-Aldrich, St Louis MO, USA) was microinjected bilaterally into the dHip at 4 μg per side. Ethanol injections were administered i.p. at a concentration of 1.5 g kg^−1^ (20% v/v) and brains were harvested 15 min post injection.

### A_2A_R and ERK-mediated impulsivity

We used DRL-controlled Pavlovian-and-operant conditioning to measure impulsive goal-tracking and sign-tracking impulsivity,^26, 27^ as described below. Detailed methodology is provided in the Supplementary information.

### Experiment 1: A_2A_R inhibition and DRL-conditioning

To examine the effects of A_2A_R antagonism on impulsivity C57BL/6J mice were administered (i.p.) ZM-241385 or vehicle 2 h prior to testing.

### Experiment 2: A_2A_R inhibition and non-contingency training

To ascertain the effect of A_2A_R inhibition in the absence of conditioning, C57BL/6J mice were administered (i.p.) ZM-241385 or vehicle 2 h prior to testing.

### Experiment 3: U0126 and DRL-conditioning

To test the effects of ERK1/2 inhibition on impulsive sign-tracking, U0126 or vehicle was bilaterally infused (4 μg μl^−1^ per side) into the dHip of C57BL/6J mice 30 min prior to testing at a rate of 0.5 μl min^−1^ for a total of 2 min. Injections were allowed to diffuse for an additional 2 min before removal of injectors, as described below.

### 5-CSRTT

Next, we explored how context predictability and A_2A_R antagonism affected 5-CSRTT performance.^28^ The trials of this task were modified so that the internal-trial intervals (INT-TI) immediately preceding aperture illumination had either random (rINT-TI; unpredictable context) or fixed (fINT-TI; predictable context) durations. Detailed 5-CSRTT methodology is provided in the Supplementary Information.

### Experiments 4–5: A_2A_R inhibition and context predictability

In these experiments, we tested whether context predictability affected impulsivity. For experiment 4 (rINT-TI) and experiment 5 (fINT-TI), C57BL/6J mice were treated with either vehicle or ZM-241385 (i.p.) 2 h prior to 5-CSRTT testing.

### Experiments 6–7: ENT1 deletion and DRL-conditioning for sucrose and sucrose–ethanol reward

To determine the effects of sucrose (20%) reward (experiment 6) or sucrose–ethanol (10%) reward (experiment 7) on goal-tracking impulsivity in WT and ENT1^–/–^ mice, we utilized DRL-mediated Pavlovian-conditioning.

### Experiment 8: ENT1 deletion and second-order discrimination

Sign-tracking impulsivity during extinction was examined in WT and ENT1^–/–^ mice during a second-order discrimination.^29, 30^ Detailed methods are provided in the Supplementary Information.

### Cannulation and U0126 microinjection

Surgeries targeting the dHip (AP: −2.1 mm from Bregma; lateral: ±1.8 mm and DV: −1.6 mm below the dural surface)^31^ for microinjection with U0126 (4 μg μl^−1^ per side at 0.5 μl min^−1^ for 2 min.) were performed as previously described.^32^ Following cannulation surgery, mice were allowed to recover for 6–7 days before starting food restriction. To ensure the health of our subjects during conditioning following cannulation surgeries, we used their pre-surgery ad libitum feeding weight to calculate their post-surgery target weight. Vehicle and U0126 microinjections were performed ~30 min prior to Pavlovian-conditioning testing. Detailed methods are available in the Supplementary Information.

### Western blot

Western blot analysis of the dHip and NAc of behavior-naive and behavior-tested ENT1^–/–^ mice, WT mice, and ZM-241385 or vehicle-treated C57BL/6J mice was performed according to standard methodology and as previously described.^33^ Briefly, the dHip and NAc from each mouse were homogenized in a Storm 24 magnetic Bullet Blender for 4 min at a speed setting of 4 (Next Advance, Averill Park, NY, USA), with 0.5 mm zirconium oxide beads in combination with Cell-lytic MT mammalian tissue extraction buffer (Sigma-Aldrich). Protein concentration from each biological replicate was quantified and replicates were loaded at 30 μg where they were separated via SDS-PAGE on a 4–12% Nu-Page Bis-Tris gel (Invitrogen, Carlsbad, CA, USA), followed by transfer to a PVDF membrane (Invitrogen). Samples were then immunoblotted overnight at 4 °C with primary antibodies specific for the A_2A_R (1:500), ERK1/2 (1:500), phospho-ERK (pERK) 1 and pERK2 (Thr-202 and Tyr-204, respectively; 1:500), GAPDH (1:1000) and appropriate secondary antibodies. Detailed methods are available in the Supplementary Information.

### Immunofluorescence and stereological analysis

We examined expression of markers indicative of cell and neuroblast proliferation (BrdU^+^, MCM2^+^ and DCX^+^) from the whole hippocampal DG (5 sections per brain, 200 μm apart from anterior to posterior) in age-matched (6-week old) behavior-naive WT and ENT1^–/–^ mice (*n*=4–5 per genotype). Similarly, we examined the process of neuroblast proliferation (BrdU^+^DCX^+^) in 5-CSRTT-tested mice administered with ZM-241385 or vehicle (*n*=5–6 per treatment). From these behavior-tested mice, we investigated the whole hippocampus (5 sections per brain, 200 μm apart from anterior to posterior) and separately the dHip (3 sections per region, 200 μm apart from anterior to posterior) and ventral hippocampus (vHip; 3 sections per region, 200 μm apart from anterior to posterior) to assess region-specific differences. Sample volumes were determined from these sections and cell density was multiplied by the total volume to yield the absolute cell numbers.^34, 35^Coronal brain sections (40 μm thick) from each mouse brain were processed to label proliferating cells with primary antibodies for BrdU^+^ (1:250), MCM2^+^ (BM28;1:500) and neuroblasts in the stage of maturation where doublecortin (DCX^+^;1:500) is expressed. Appropriate secondary antibodies (Cy2, Cy3 and Cy5) were used to detect primary antibodies.^34, 36^ Images were acquired on a LSM 780 confocal system (Zeiss, Carl Zeiss Microscopy, Thornwood, NY, USA) with × 20 and × 40 objectives using a multi-tile configuration. Stereological quantification of BrdU^+^, BrdU^+^DCX^+^ and BrdU^+^MCM2^+^ cells within the subgranular zone (SGZ), and granule cell layer of the DG were carried out using Zen Blue edition (Zeiss) as previously described.^34, 35^

### Data and statistical analysis

Detailed statistical analyses can be found in the Supplementary Information.

Pavlovian-conditioning and non-contingency testing: For behavioral analysis, we conducted repeated measures two-way ANOVA (RM two-way ANOVA), two-way ANOVA and unpaired two-tailed Student's *t*-test as described in detail in the Supplementary Information. Discrimination: For behavioral analysis of second-order discrimination, we used two-way ANOVA. 5-CSRTT: Behavioral comparisons were analyzed with RM two-way ANOVA and unpaired two-tailed Student's *t*-test. Open field: Velocity and distance traveled were analyzed with RM two-way ANOVA followed by Bonferroni's multiple comparisons, where appropriate.

For western blot analysis, each lane represents an individual brain region biological replicate that was normalized to its respective GAPDH protein expression. We utilized an unpaired Student's *t*-test or one-way ANOVA for western blot comparisons which is described in detail in the Supplementary Information. Images are representative of 1–3 western blotting experiments. For all BrdU^+^, BrdU^+^MCM2^+^ and BrdU^+^DCX^+^ statistical analyses, cell numbers derived from 3–5 coronal sections were averaged for each brain, and we utilized an unpaired Student's *t*-test for comparisons. Statistical significances reported for all ANOVA results were followed with Tukey's multiple comparisons where appropriate (Sigma Plot 12.0, Systat Software, San Jose, CA, USA). Results were considered statistically significant when *P*<0.05 and are presented as mean and ±s.e.m. (GraphPad Prism, La Jolla, CA, USA).

## Results

### A_2A_R Inhibition induces impulsivity when delivery of reward is unpredictable

**Figure 1:**
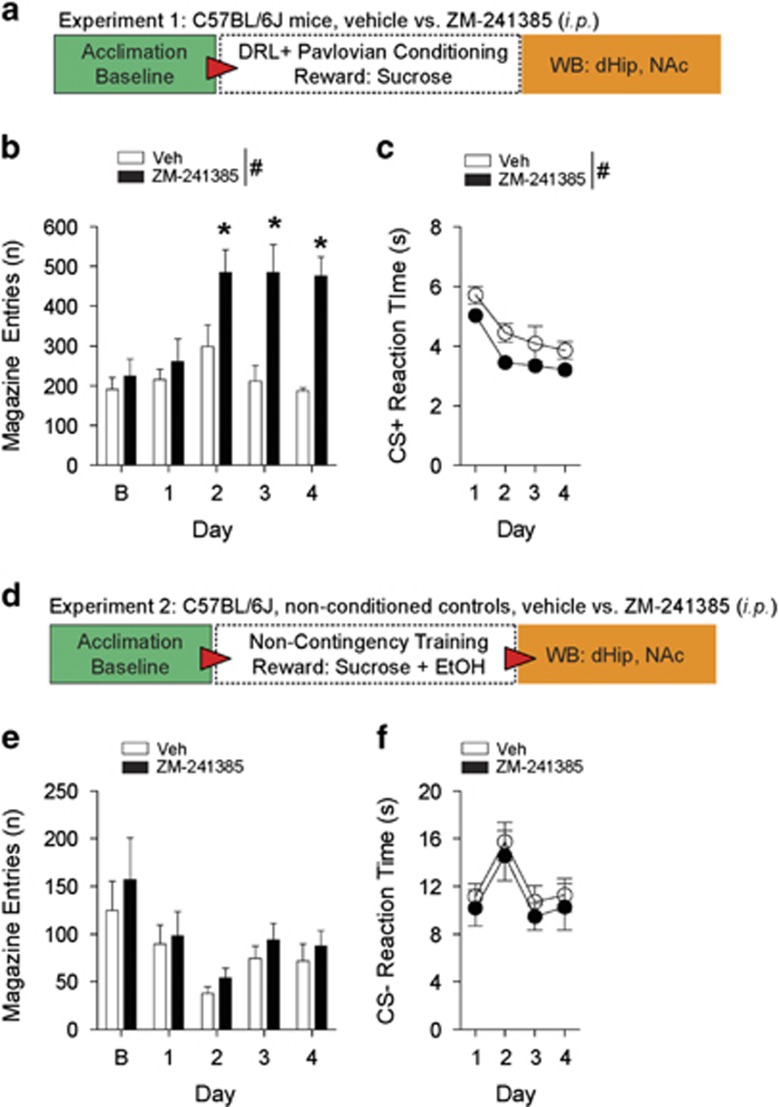
Pharmacological inhibition of the A_2A_R promotes impulsivity when reward is unpredictable during DRL-conditioning. (**a**) Schematic of experimental design for testing impulsivity during Pavlovian-conditioning. (**b**) In relation to vehicle, mice-administered ZM-241385 display significantly higher magazine entries for sucrose reward during DRL-conditioning. (**c**) Mice treated with ZM-241385 display significantly faster reaction times to CS+ presentations relative to vehicle. (**d**) Schematic of experimental design for testing impulsivity during non-contingency training. (**e**) There were no differences detected in magazine entries between mice treated with ZM-241385 and vehicle-treated mice during non-contingency training. (**f**) During non-contingency training, mice treated with ZM-241385 or vehicle did not display differences in reaction times to CS-presentations. All data are expressed as mean±s.e.m. RM two-way ANOVA, ^#^*P*<0.05 main effect of treatment (i.p.); *P<0.05 by Tukey's *post hoc* analysis versus vehicle-treated mice. (**b**, **c** *n*=5–6 per treatment; **e**, **f** *n*=10 per treatment). DRL, differential reward of low rate; RM, repeated measures.

To assess the contribution of the A_2A_R to impulsivity during DRL-conditioning (Figure 1a), mice were required to wait for CS+ presentations for reward delivery.^37, 38^ As demonstrated by increased magazine entries, mice administered (i.p.) the specific A_2A_R antagonist ZM-241385 (Figure 1b) displayed exacerbated impulsivity during conditioning (F_(1,9)_=17.62, *P*<0.01) on days 2–4 (*P*<0.05) and exhibited faster approach reaction times (Figure 1c) in response to CS+ (F_(1,9)_=7.69, *P*<0.05). Both groups equally learned the conditioning task as reaction times significantly decreased (F_(3,27)_=23.10, *P*<0.001). There were no differences in inactive-hole entries (Supplementary Figure 2a). Conversely, when rewards were not contingent on waiting for CS-presentations and rewards were predictable (Figure 1d), ZM-241385 failed to induce impulsive magazine entries (Figure 1e) in control mice (F_(1,18)_=1.34, *P*=0.261). Moreover, Figure 1f shows that pharmacological treatment had no effect on CS- reaction times (F_(1,18)_=0.75, *P*=0.398). Inactive-hole entries in this control experiment were not different between treatment groups (Supplementary Figure 2b).

### Inhibition of ERK1/2 in the dHip potentiates impulsivity

**Figure 2:**
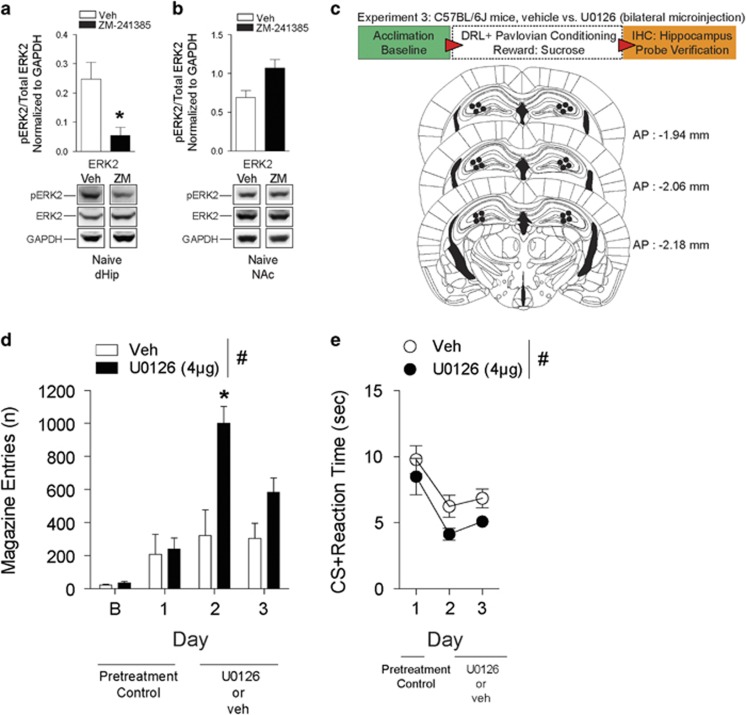
Lowered ERK1/2 phosphorylation in the dorsal hippocampus (dHip) exacerbates impulsivity during DRL-conditioning. (**a**) Western blot analysis in the dHip revealed a significant decrease in pERK2 in mice naive to behavior testing and treated with ZM-241385 versus vehicle. *n*=3 per treatment (i.p.). *P<0.05 by Student's *t*-test. (**b**) There were no differences in pERK2 expression in the nucleus accumbens (NAc) of vehicle and ZM-241385-treated mice that were naive to behavior testing. *n*=3 mice per treatment (i.p.). *P<0.05 by Student's *t*-test. (**c**) Schematic of experimental design for testing impulsivity during Pavlovian-conditioning and dHip microinjection-targeting coordinates. (**d**) Mice bilaterally infused with U0126 into the dHip display significantly higher magazine entries for sucrose reward during conditioning relative to vehicle. (**e**) Mice bilaterally infused with U0126 display significantly faster reaction times to CS+ presentations during conditioning. All data are expressed as mean±s.e.m. RM two-way ANOVA, ^#^*P*<0.05 main effect of treatment; *P<0.05 by Tukey's *post hoc* analysis versus vehicle-treated mice (**d**, **e** *n*=3–4 per treatment). DRL, differential reward of low rate; RM, repeated measures.

Hippocampal A_2A_R function is known to modulate ERK1/2 activity.^17^ In agreement, our western blot analysis revealed that in comparison to vehicle, naive mice administered ZM-241385 had significant decreases of pERK1 (*t*_4_=3.07, *P<0.05) and pERK2 (*t*_4_=3.06, *P<0.05) in the dHip (Figure 2a and Supplementary Figure 8a) but not in the NAc (Figure 2b and Supplementary Figure 9a). Thus, we investigated whether direct inhibition of hippocampal pERK1/2 with the selective MEK inhibitor U0126 would affect impulsivity (Figure 2c). Microinjections inhibiting ERK1/2 in the dHip (Figure 2d) 30 min prior to DRL-conditioning significantly potentiated (F_(1,15)_=27.72, *P*<0.01) impulsive magazine entries (day 2, *P<0.001). Although both treatment groups exhibited decreased CS+ reaction times (F_(2,15)_=10.99, *P*<0.01), mice administered U0126 relative to vehicle consistently displayed faster CS+ elicited reaction times (F_(1,15)_=5.56, *P*<0.05) (Figure 2e). Inactive-hole entries (Supplementary Figure 2c) were not different between treatment groups (*t*_5_= 2.16, *P*=0.08). These results suggest that inhibition of pERK1/2 in the dHip has a role in the potentiation of impulsive goal-tracking behavior.

### A_2A_R inhibition potentiates 5-CSRTT sign-tracking impulsivity when reinforcement is unpredictable

**Figure 3:**
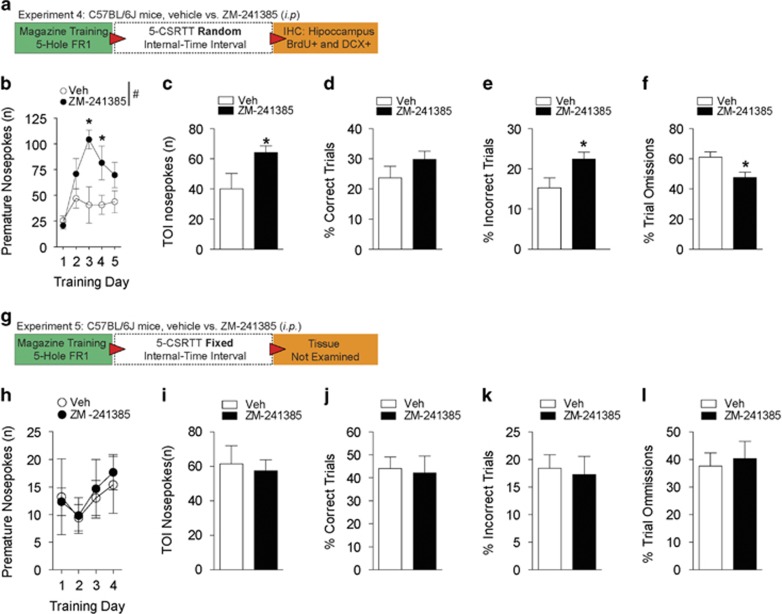
Unpredictability of reward delivery and A_2A_R antagonism exacerbates impulsivity during the 5-CSRTT. (**a**) Schematic of experimental design for testing impulsivity during the 5-CSRTT under a random internal trial-interval (rINT-TI). (**b**) Mice administered (i.p.) ZM-241385 and subjected to rINT-TI aperture illuminations, display significantly higher impulsive premature nosepokes. *P<0.05 by Student's *t*-test. (**c**) Mice administered ZM-241385 and subjected to a rINT-TI show significantly higher impulsive TOI nosepokes. *P<0.05 by Student's *t*-test. (**d**) Both treatment groups performed the 5-CSRTT equally well, as our analysis did not detect a difference in the percentage of correct trials completed. (**e**) An examination of the percentage of incorrect trials revealed that mice administered ZM-241385 had significantly higher errors during 5-CSRTT performance. *P<0.05 by Student's *t*-test. (**f**) Mice treated with ZM-241385 had a significantly lower percentage of omissions during the 5-CSRTT. (**g**) Schematic of experimental design for testing impulsivity during the 5-CSRTT under a fixed internal trial-interval (fINT-TI). (**h**) Mice administered ZM-241385 did not show differences in premature nosepokes during fINT-TI 5-CSRTT performance. (**i**) Mice administered ZM-241385 or vehicle did not display differences in TOI nosepokes during fINT-TI 5-CSRTT performance. (**j**) There were no differences between the treatment groups in the percentage of correct trials completed during fINT-TI 5-CSRTT testing. (**k**) There were no differences between the treatment groups in the percentage of incorrect trials completed during fINT-TI 5-CSRTT testing. (**l**) There were no differences between the treatment groups in the percentage of omission trials during fINT-TI 5-CSRTT testing. All data are expressed as mean±s.e.m. (**b–f** *n*=5–6 per treatment; **h–l** *n*=5–6 per treatment) RM two-way ANOVA, ^#^*P*<0.05 main effect of treatment; *P<0.05 by Tukey's *post hoc* multiple comparisons versus vehicle-treated mice. 5-CSRTT, 5-choice serial reaction time task; DRL, differential reward of low rate; RM, repeated measures; TOI, time-out interval.

Next, we asked whether A_2A_R inhibition and cue predictability induced sign-tracking impulsivity. Prior to 5-CSRTT testing, mice underwent magazine training and 5-hole FR-1 operant conditioning (Supplementary Figures 3a–c). When waiting times for reinforcement-associated cues were unpredictable (random internal time interval, rINT-TI; Figure 3a) during 5-CSRTT testing, mice that were administered i.p. ZM-241385 (Figure 3b) emitted significantly more premature nosepokes (F_(1,9)_=6.60, *P*<0.05) during training (days 3–4, *P*<0.05). Likewise, A_2A_R inhibition resulted in significantly more impulsive time-out interval nosepokes (*t*_9_=2.33, *P*<0.05; Figure 3c). There were no differences in the proportion of correct trials (Figure 3d) or magazine entries (Supplementary Figure 3e). As shown in Figures 3e–f, mice administered ZM-241385 displayed a significantly higher proportion of incorrect trials (*t*_9_=2.45, *P*<0.05) and a lower percentage of omissions (*t*_9_=2.68, *P*<0.05). Conversely, when waiting times for reinforcement-associated cues were predictable (fixed INT-TI; Figure 3g), A_2A_R inhibition did not increase impulsive premature nosepokes (Figure 3h). In comparison to vehicle treatment, ZM-241385 did not significantly affect time-out interval nosepokes, magazine entries, or the proportion of correct trials, incorrect trials and trial omissions (Figures 3i–l and Supplementary Figure 3f).

Previous reports indicate that ZM-241385 increases locomotor activity in rodents,^39, 40^ thus we examined whether A_2A_R inhibition in combination with food restriction (which can itself increase locomotion) affected ambulatory velocity and distance traveled.^41^ Although both i.p. vehicle and ZM-241385-treated mice significantly decreased ambulatory velocity (F_(17,306)_=4.06, *P*<0.0001), we did not detect an interaction or a significant difference in velocity between treatment groups (Supplementary Figure 4a). In agreement with previous reports, our analysis detected more distance traveled in mice treated with ZM-241385 ((F_(1,18)_=4.56, *P*<0.05); *P<0.05 at 120 min and 150 min) in comparison to vehicle (Supplementary Figure 4b). A significant effect of time post injection (F_(17,306)_=2.22, *P*<0.01) and an interaction between treatment and time post injection (F_(17,306)_=3.02, *P*<0.001) was also detected (Supplementary Figure 4b). Taken together, our DRL-conditioning and 5-CSRTT results provide evidence to indicate that A_2A_R antagonism increases impulsivity and shortens reaction times in contexts where reward or reinforcement delivery is unpredictable, without significantly affecting locomotor velocity.

### ENT1^–/–^ mice display increased impulsivity, decreased hippocampal A_2A_R expression and lowered ERK phosphorylation

**Figure 4:**
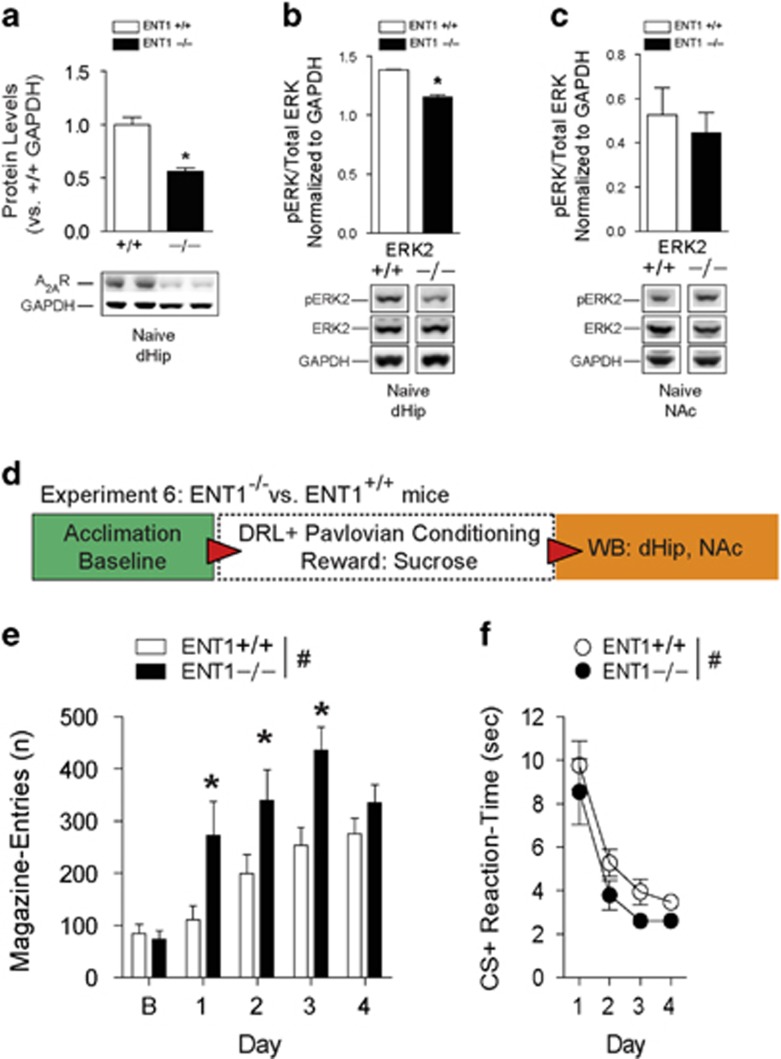
Mice lacking ENT1 display aberrant impulsivity during DRL-conditioning and exhibit dampened dorsal hippocampus (dHip) A_2A_R expression and lowered ERK1/2 phosphorylation. (**a**) Western blot and densitometry quantitation show significantly decreased expression of A_2A_R in the dHip of behavior-naive ENT1^–/–^ mice versus WT mice. *n*=11 mice per genotype. *P<0.05 by Student's *t*-test. (**b**) Behavior naive ENT1^–/–^ mice have decreased pERK2 in the dHip relative to WT mice. *n*=3 per genotype. *P<0.05 by Student's *t*-test. (**c**) There were no differences in pERK2 in the nucleus accumbens (NAc) of naive WT and ENT1^–/–^ mice. *n*=4 mice per genotype. (**d**) Schematic of experimental design for testing impulsivity during Pavlovian-conditioning in ENT1^–/–^ mice versus WT mice. (**e**) ENT1^–/–^ mice display significantly higher magazine entries for sucrose reward during conditioning. (**f**) Relative to WT mice, ENT1^–/–^ mice display faster reaction times to retrieve sucrose reward in response to CS+. All data are reported as mean±s.e.m. RM two-way ANOVA, ^#^*P*<0.05 main effect of genotype; *P<0.05 by Tukey's *post hoc* multiple comparisons versus WT mice. (**e**, **f** *n*=13–15 per genotype). DRL, differential reward of low rate; RM, repeated measures; WT, wild-type.

We have previously reported that decreased functionality of ENT1 and striatal A_2A_R have a critical role in goal-oriented reward seeking.^19^ Given that our study and others indicate involvement of the hippocampus during reward-mediated prediction error behavior,^42^ our western blot results in the dHip of behavior-naive ENT1^–/–^ mice revealed a significant decrease (*t*_20_ =4.90, *P*<0.001) in A_2A_R expression (Figure 4a and Supplementary Figure 8b). This signature was not observed in the NAc (Supplementary Figure 9b). We also detected a significant decrease in pERK2 (*t*_4_=10.45, *P<0.001), although not pERK1 in the dHip of behavior-naive ENT1^–/–^ mice (Figure 4b and Supplementary Figure 8c). An analysis of the NAc in behavior-naive ENT1^–/–^ mice did not identify a significant difference in pERK1 or pERK2 (Figure 4c and Supplementary Figure 9c) in comparison to WT mice. Next, we tested whether ENT1^–/–^ mice would display impulsive goal-tracking during Pavlovian-conditioning (Figure 4d). Accordingly (Figure 4e), ENT1^–/–^ mice displayed significantly higher magazine entries for sucrose reward (F_(1,26)_=8.45, *P*<0.01) on training days 1–3 (all *P*<0.05). Both genotypes increased impulsive magazine entries during training (F_(4,104)_=22.21, *P*<0.001), and a genotype and training interaction (F_(4,104)_=3.24, *P*<0.05) was detected. Further, both genotypes learned the conditioning task as evidenced by decreased reaction times in response to CS+ (F_(3,78)_=28.35, *P*<0.001), with ENT1^–/–^ mice exhibiting significantly faster (F_(1,26)_=4.01, *P*<0.05) CS+ elicited reaction times (Figure 4f). Inactive-hole entries were not significantly different between the genotypes (Supplementary Figure 5a). Reaffirming ethanol's well-known association with impulsivity^43^ (Supplementary Figures 5b,c), both genotypes displayed increased impulsive goal-tracking for ethanol reward (F_(4,52)_=16.88, *P*<0.001). Not surprisingly, ENT1^–/–^ mice displayed excessively higher magazine entries (F_(1,13)_=12.13, *P*<0.01) on days 1–3 (all *P*<0.05) in comparison to WT (Supplementary Figure 5c). A significant genotype and training day interaction (F_(4,52)_=3.53, *P*<0.01) was also detected. In addition, both genotypes (Supplementary Figure 5d) decreased CS+ reaction times as conditioning was acquired (F_(3,39)_=20.64, *P*<0.001), with ENT1^–/–^ animals again displaying significantly faster reaction times (F_(1,13)_=6.81, *P*<0.05). There were no genotype differences in inactive-hole entries (Supplementary Figure 5e).

These results suggest that deficits in ENT1-adenosine signaling may regulate impulsivity, and further demonstrate that consumption of highly hedonic sweetened alcohol drinks can promote dangerously pathological seeking behavior.^44^ This becomes evident when comparing the exacerbated impulsive goal-tracking behavior for sucrose–ethanol reward relative to sucrose reward (Supplementary Figures 6a and b), exhibited by both WT (*t*_22_=3.06, *P*<0.01) and ENT1^–/–^ mice (*t*_17_=4.31, *P*<0.001).

### ENT1^–/–^ mice display sign-tracking impulsivity

Next, we explored whether ENT1^–/–^ mice displayed sign-tracking impulsivity during an operant second-order discrimination task (Supplementary Figure 7a). This paradigm is used to investigate cue-induced self-administration of cocaine,^45^ heroin^46^ and alcohol.^47^ As shown in Supplementary Figure 7b, we did not detect genotype differences in active-hole nosepokes during FR-1 pretraining. As both genotypes learned the operant, trial completions increased (F_(3,54)_=3.81, *P*<0.05), although there was no difference in completions between WT and ENT1^–/–^ mice (Supplementary Figure 7c). Both genotypes demonstrated an overall extinction decrease (F_(3,54)_=6.88, *P*<0.01) of ITI nosepokes (Supplementary Figure 7d). As expected, ENT1^–/–^ mice emitted significantly higher nosepokes (F_(1,54)_=4.91, *P*<0.05) during the ITI period. Overall, discrimination performance improved for both genotypes (F_(3,54)_=7.02, *P*<0.01), although discrimination performance was worse in ENT1^–/–^ mice compared to WT (F_(1,54)_=4.67, *P*<0.05), as evidenced by a significantly lower discrimination ratio (Supplementary Figure 7e).

### ERK1/2 phosphorylation in the dorsal hippocampus is increased following DRL-conditioning

ERK activity is highly dynamic, often changing as a result of behavioral manipulations and drugs of abuse.^48, 49, 50^ Thus, we examined the effects of repeated days of impulsive Pavlovian-conditioning on phosphorylation of this kinase. In comparison to vehicle, our analysis detected significantly higher pERK1 (*t*_6_=2.63, *P*<0.05) and pERK2 (*t*_6_=3.04, *P*<0.05) in the dHip of mice administered ZM-241385 which underwent behavior conditioning (Supplementary Figure 8a), without similar changes in the NAc (Supplementary Figure 9a). Notably, pERK1 or pERK2 alterations were not detected in the dHip following non-contingency training (Supplementary Figure 10c), suggesting that unpredictability of reward during waiting impulsivity has a unique role in altered hippocampal ERK signaling. An analysis of ERK signaling in the NAc following non-contingency training detected a higher ratio of pERK1 (*t*_6_=3.39, *P*<0.05) but not pERK2 in the NAc of mice treated with ZM-241385, likely due to lower expression of total ERK1 (Supplementary Figure 10b).

Our results indicate that ENT1^–/–^ mice exhibit increased impulsivity. Consequently, we examined ERK1/2 signaling in the dHip and NAc. Our results show that relative to WT mice, ENT1^–/–^ mice exhibited significantly higher pERK1 (*t*_4_=6.15, *P*<0.01) and pERK2 (*t*_4_=3.82, *P*<0.05) in the dHip (Supplementary Figure 8c) but not the NAc (Supplementary Figure 9c), following behavior conditioning. Although we report increased pERK1/2 following conditioning for sucrose, we were surprised to find that ENT1^–/–^ mice, which displayed exacerbated impulsive goal-tracking for sucrose–ethanol reward, did not have a similar signature of pERK1 and pERK2 in the dHip (Supplementary Figure 11a). Therefore, we confirmed that relative to vehicle injection (F_(3,11)_=62.59, *P*<0.0001), ethanol (1.5 g/kg i.p.) significantly lowers pERK1 (*P*<0.01) and pERK2 (*P*<0.001) in the dHip of WT mice (Supplementary Figure 11b). Interestingly, others studies have also shown ethanol-induced reductions in hippocampal ERK1/2 activity.^51^ Ethanol injection also had a similar effect in the dHip of ENT1^–/–^ mice (F_(3,11)_=21.27, *P*<0.001) as phospho-ERK1 (*P*<0.01) and phospho ERK2 (*P*<0.01) was significantly dampened in comparison to vehicle (Supplementary Figure 11b). Thus, when compared to a natural reward (sucrose), our results potentially provide a novel explanation of how ethanol's effect on ERK phosphorylation may be associated with ethanol-induced exacerbations in impulsive behavior (Supplementary Figure 6).

Next, we examined the change of pERK1 and pERK2 signaling between behavior-naive animals in relation to mice that underwent DRL-conditioning and received ZM-241385 or vehicle. As observed in Supplementary Figures 8a and 12a, pERK1 and pERK2 in the dHip of vehicle-treated mice was not significantly different as a result of DRL-conditioning. In contrast, pERK1 (*t*_5_=16.24, *P*<0.0001) and pERK2 (*t*_5_=12.36, *P*<0.0001) in the dHip of ZM-241385-treated mice was significantly higher as a result of DRL-conditioning (Supplementary Figures 8a and 12b). Interestingly, analysis of the NAc from vehicle-treated mice failed to detect differences in pERK1 and pERK2 resulting from DRL-conditioning (Supplementary Figures 9a and 12c). However, we did detect a significant decrease in pERK1 (*t*_4_=3.79, *P*<0.05) and pERK2 (*t*_4_=4.27, *P*<0.05) in DRL-conditioned mice that were administered ZM-241385, relative to ZM-241385-treated behavior-naive mice (Supplementary Figures 9a and 12d). These results likely stem from lower total ERK1 and total ERK2 expression for these comparisons (Supplementary Figure 9a).

A similar analysis in the dHip of WT mice revealed that both pERK1 (*t*_5_=5.10, *P*<0.01) and pERK2 (*t*_5_=10.01, *P*<0.001) were significantly decreased as a result of DRL-conditioning (Supplementary Figures 8c and 12e). In contrast, phospho-ERK1 (*t*_4_=3.04, *P*<0.05) was significantly increased in the dHip of ENT1^–/–^ mice as a result of DRL-conditioning, but not pERK2 (Supplementary Figures 8c and 12f). An examination of this change in the NAc did not reveal significant differences in pERK1 and pERK2 between behavior-naive WT mice and DRL-conditioned WT mice (Supplementary Figures 9c and 12g). Notably, we did detect a significant increase in pERK1 (*t*_7_=2.91, *P*<0.05) but not pERK2, between behavior-naive ENT1^–/–^ mice and DRL-conditioned ENT1^–/–^ mice (Supplementary Figures 9c and 12h). Taken together, these results again suggest that DRL-mediated impulsivity has a significant effect on phosphorylation of ERK1/2 primarily in the dHip, as ERK1/2 phosphorylation in the NAc was not readily apparent based on these results (Supplementary Figures 9a and c).

### Hippocampal neuroblast proliferation is dampened in ENT1^–/–^ mice

**Figure 5:**
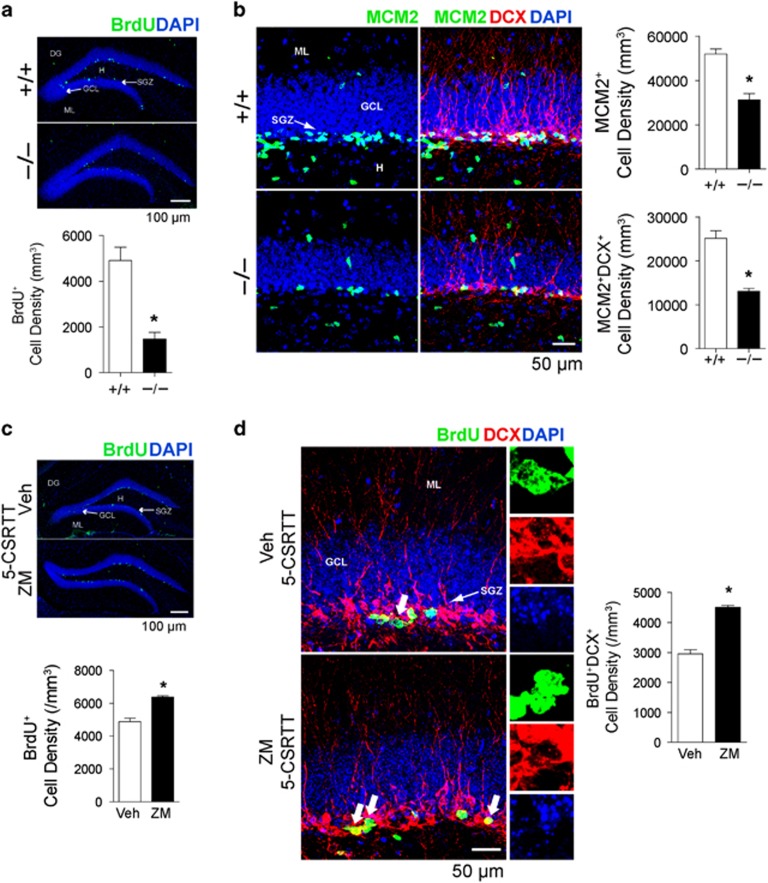
Expression of markers for cell proliferation and neuroblast development in the hippocampal dentate gyrus (DG) is affected by ENT1 deletion and A_2A_R inhibition-mediated impulsivity. (**a**) Representative confocal microscopy images and stereological quantification indicate that relative to WT, naive ENT1^–/–^ mice have decreased numbers of BrdU^+^-labeled cells (green). DAPI (blue). Scale bar, 100 μm. (**b**) Relative to WT, naive ENT1^–/–^ mice have decreased numbers of MCM2^+^ (green) DCX^+^ (red)-labeled cells. DAPI (blue). Scale bar, 100 μm. All data are reported as mean±s.e.m. (**a**, **b**) *n*=5 brain sections per brain and *n*=4–5 mice per genotype. *P<0.01 by unpaired two-tailed Student's *t*-test. (**c**) Analysis of BrdU^+^-labeled cells (green) in 5-CSRTT-tested mice indicate overall higher numbers of BrdU^+^ cells in mice administered ZM-241385 relative to vehicle. DAPI (blue). Scale bars, 100 μm. (**d**) Representative confocal microscopy images and stereological quantification indicate that in comparison to vehicle, mice administered ZM-241385 that displayed increased impulsivity during the 5-CSRTT exhibited higher numbers of BrdU^+^ (green) DCX^+^ (red) co-labeled cells. Thick arrows indicate representative individual BrdU^+^, DCX^+^ and DAPI co-labeled cells (see insets). Thin arrows indicate the subgranular zone (SGZ) or the DG of the hippocampus. Scale bars, 100 μm. (**c**, **d**) *n*=5–6 sections per brain and *n*=5–6 mice per treatment. *P<0.001 by unpaired two-tailed Student's *t*-test. All data are reported as mean±s.e.m. 5-CSRTT, 5-choice serial reaction time task; DRL, differential reward of low rate; GCL, granular cell layer; H, hilus; ML, molecular layer; RM, repeated measures; WT, wild-type.

Chronic ethanol has been shown to severely disrupt cell proliferation in the SGZ of the DG.^52^ Given our reported association between ENT1 deletion and lower pERK1/2, in addition to the reported relationship between ERK and adult neurogenesis,^53, 54^ we investigated whether ENT1^–/–^ mice displayed altered expression for markers of hippocampal cell proliferation and neuroblast development. As shown in Figure 5a, ENT1^−/−^ mice contain significantly fewer BrdU^+^-labeled cells within the SGZ of the DG, relative to WT (*t*_7_=4.86, *P*<0.01). Moreover, we detected decreased numbers of MCM2^+^ (*t*_7_=5.48, *P*<0.01), an endogenous marker of cell cycle progression and MCM2^+^DCX^+^ (*t*_7_=7.24, *P*<0.01)-labeled cells in ENT1^–/–^ mice, suggesting impairments in cell proliferation and neuroblast development resulting from ENT1 deletion (Figure 5b).

### A_2A_R inhibition-mediated impulsivity increases expression of markers for hippocampal neuroblast proliferation

This report demonstrates that A_2A_R inhibition can potentiate impulsivity, which has an effect of increasing ERK1/2 activity in the dHip. Given that ERK1/2 is associated with hippocampal neuroblast development, we sought to examine whether A_2A_R inhibition is associated with the neuroblast proliferation. Remarkably, the number of BrdU^+^-labeled cells (Figure 5c; *t*_9_=7.49, *P*<0.001) and BrdU^+^DCX^+^ co-labeled cells (Figure 5d) within the DG of ZM-241385-treated animals that exhibited increased impulsivity was significantly higher compared to vehicle (*t*_9_=11.20, *P*<0.001). More importantly, a region-specific analysis identified that the increased neuroblast proliferation resulting from A_2A_R inhibition occurs primarily in the dHip as BrdU^+^ expression (*t*_9_=3.94, *P*<0.01) and BrdU^+^DCX^+^ co-labeling (*t*_9_=2.42, *P*<0.05) was significantly higher in the dHip (Supplementary Figures 13a and b), but not in the ventral hippocampus (vHip) (Supplementary Figures 13a and c).

## Discussion

Drugs of abuse promote maladaptive risk taking for the pursuit of immediate gratification although functionally impulsive risk taking confers an evolutionary advantage by encouraging the pursuit of unexpected opportunities.^55, 56^ In this study, we demonstrate that pharmacological inhibition of the adenosine A_2A_R and ERK1/2 exacerbates waiting impulsivity, an essential predictor of alcohol use disorders.^57^ More importantly, our results show an association of A_2A_R antagonist-mediated increases in impulsivity and hippocampal neuroblast proliferation.

Although A_2A_R hypofunction enhances synaptic plasticity and memory function,^16, 58, 59^ hippocampal A_2A_R activation is implicated in memory impairment and cognitive decline.^17^ Our results indicate that A_2A_R hypofunction in combination with heightened states of reward seeking can maladaptively exacerbate impulsive behavior in contexts, where reward gratification is unpredictable. Supporting this, A_2A_R antagonism failed to induce impulsive goal-tracking when reward gratification was not contingent on acquisition of conditioning. Similarly, when cue-signaled reinforcement was predictable during the 5-CSRTT, ZM-241385 did not induce impulsive sign-tracking responses. It is possible that inhibition of the A_2A_R, which includes the effects of caffeine, may mediate increases in locomotion,^40, 60, 61^ which when in combination with reward/reinforcement unpredictability, leads to compounded exploratory activity and exacerbations in impulsive reward-seeking behavior. Therefore, we posit that dysfunctional adenosinergic regulation can exacerbate the inherent cognitive dissonance between the drive for reward gratification and a reward, which may or may not be available.^62^

Validating these observations, the alcohol preferring ENT1^–/–^ mice demonstrated excessive goal-tracking impulsivity and faster reaction times in response to CS+, traits indicative of inadequate self-control reported in addiction.^6, 63^ Moreover, we revealed lowered A_2A_R expression and decreased pERK1 (Thr-202) and pERK2 (Tyr-204) in the dHip of behavior-naive ENT1^–/–^ mice. Interestingly, a similar decrease in pERK1/2 was observed in the dHip of behavior-naive mice following A_2A_R antagonism. Several lines of evidence suggest that inhibition of ERK1/2 can increase behavioral excitement, decrease depressive-like symptomatology and produce marked hyperactivity.^22, 64, 65^ In agreement, direct infusion of the MEK inhibitor U0126 into the dHip increased goal-tracking impulsivity. The ability for ethanol to significantly dampen hippocampal pERK1 and pERK2, whereas notably exacerbating impulsivity strengthens our findings. Interestingly, the hippocampus-specific contribution of pERK1 and pERK2 to the regulation of impulsivity was underscored by the comparatively stable phosphorylation levels of these kinases within the NAc of naive and DRL-conditioned ENT1^–/–^ mice as well as ZM-241385-treated mice. This finding is surprising because patients with attention-deficit hyperactivity disorder and alcoholism have been demonstrated to exhibit hypoactivation of the ventral striatum during impulsive behavior.^7, 66^ Conversely, increased ventral striatal activity has been observed in normal individuals during impulsive performance.^67^ Furthermore, recent *in vivo* electrophysiological studies examining accumbal activation of local field potentials during impulsive performance of the 5-CSRTT suggest that this brain region is activated during anticipation of cue-signaled reinforcement, movement to retrieve reinforcement, as well as during consumption of reinforcers.^68^ One possibility for our inability to detect more obvious ERK1 and ERK2 phosphorylation changes in the NAc is that our results are derived from auditory CS+-controlled Pavlovian approach responses. This is in contrast to 5-CSRTT operant behavior, where visual cues control nosepoking behavior. Therefore, given the complexity of the overlapping cortical, striatal, sub-thalamic and amygdalar neural substrates, the molecular regulation of waiting versus decisional impulsivity may be differentially affected by behavioral task (that is, DRL versus delay discounting).^27, 69, 70^

We and other investigators report that behavioral conditioning, as well as pharmacological and biochemical manipulation can alter ERK activity in a region-specific manner.^71, 72, 73^ Interestingly, we also observed increased dHip pERK1 and pERK2 activity following repeated conditioning days for sucrose reward. Importantly, this increase in phosphorylation was not observed in mice that did not display impulsivity during non-contingency training, suggesting that pERK1 and pERK2 may have a biological rheostatic role adapting to risk taking behavior, much like CREB's reported function as an ‘emotional rheostat' in response to stress, anxiety and depression.^74^ Our results echo well-reported findings in cell-culture studies as well as in global and conditional knockout systems indicating that although ERK1 and ERK2 are coded from different genes, each provides a redundant role for the other in terms of cell signaling and behavior.^75^ However, alternative upstream and downstream effectors may also be having a role in affecting adenosine and ERK signaling (that is, PKA, CREB or ELK), and warrant further study.^76, 77, 78^

Impairment of hippocampal neurogenesis is known to increase vulnerability for alcohol addiction and relapse.^24, 52^ Our results elucidate a novel regulatory role for adenosinergic signaling in the process of neuronal proliferation and maturation, as ENT1^–/–^ mice exhibit a reduction of cells labeled with BrdU^+^ and DCX^+^. Conversely, A_2A_R antagonism potentiated cell proliferation and increased markers of neuroblast development in the dHip but not the vHip, suggesting a region-specific role in regulation of this process. Notably, several recent investigations have revealed a differential regulation of adult neurogenesis in terms of stress-induced depressive behavior along the dorso-ventral axis of the hippocampus.^79^ In particular, rodent studies examining stress-induced depression-like behavior and antidepressant efficacy have elucidated specific involvement of the vHip, but not the dHip, in this process.^80, 81^ Our results yield indicate that A_2A_R inhibition distinctly targets the dHip in terms of neuroblast proliferation and possibly subsequent adult neurogenesis, which seems to be associated with can enhanced impulsive reward seeking. Indeed, recent studies suggest that states of withdrawal induce craving for reward, which increase adult neurogenesis.^25, 82^ Although, we acknowledge that performance of the 5-CSRTT in this study was done while seeking a natural reward. This raises the possibility that the observed increases in cell proliferation and expression of immature neuroblasts may represent a natural process and not necessarily an aberrant process. However, we may surmise that a more potent addictive substance may aberrantly engage this process, which could lead to maladaptive drug seeking.

In summary, our results suggest that maladaptive impulsivity is potentiated by A_2A_R hypofunction and pERK1/2 downregulation in the dHip. Moreover, we demonstrate that impulsivity is associated with increases in expression of markers indicative of the neuroblast proliferation specifically in the dHip. This process, which is known to be a critical precursor to adult neurogenesis, may be regulated through elevations in ERK activity. Thus, our findings suggest a novel role for the A_2A_R in maladaptive impulsivity as well as potentially providing a new investigative avenue to examine the relationship between neurogenesis and reward seeking behaviors.
