# Supplementary material for: Adenosine A2A receptor and ERK-driven impulsivity potentiates hippocampal neuroblast proliferation
Source: Transl Psychiatry. 2017 Apr 18;7(4):e1095–. doi: 10.1038/tp.2017.64 (PMC5416704; doi:10.1038/tp.2017.64)
Supplement: Supplementary Information [file tp201764x1.pdf]

Supplementary Information

Adenosine A2A Receptor and ERK Driven Impulsivity Potentiates Hippocampal Neuroblast Proliferation

Alfredo Oliveros[1], Chang Hoon Cho[3], Allen Cui[1], Sun Choi[1], Daniel Lindberg[2], David Hinton[2], Mi-Hyeon Jang[3,4], and Doo-Sup Choi[1, 2, 5]

[1]Department of Molecular Pharmacology and Experimental Therapeutics, [2]Neurobiology of Disease Program, [3]Department of Neurologic Surgery, [4]Department of Biochemistry and Molecular Biology, [5]Department of Psychiatry and Psychology, Mayo Clinic College of Medicine Rochester, Minnesota 55905, USA.

Running Title: Adenosine$_{A2A}$-ERK1/2 Impulsivity Increases Hippocampal-Cell Proliferation

Correspondence and requests for materials:
Doo-Sup Choi, PhD
Department of Molecular Pharmacology and Experimental Therapeutics
Mayo Clinic College of Medicine
200 First Street SW. Rochester, Minnesota 55905, USA
Email: choids@mayo.edu, Fax: 507-284-1767, Telephone: 507-284-5602

Supplementary Figures

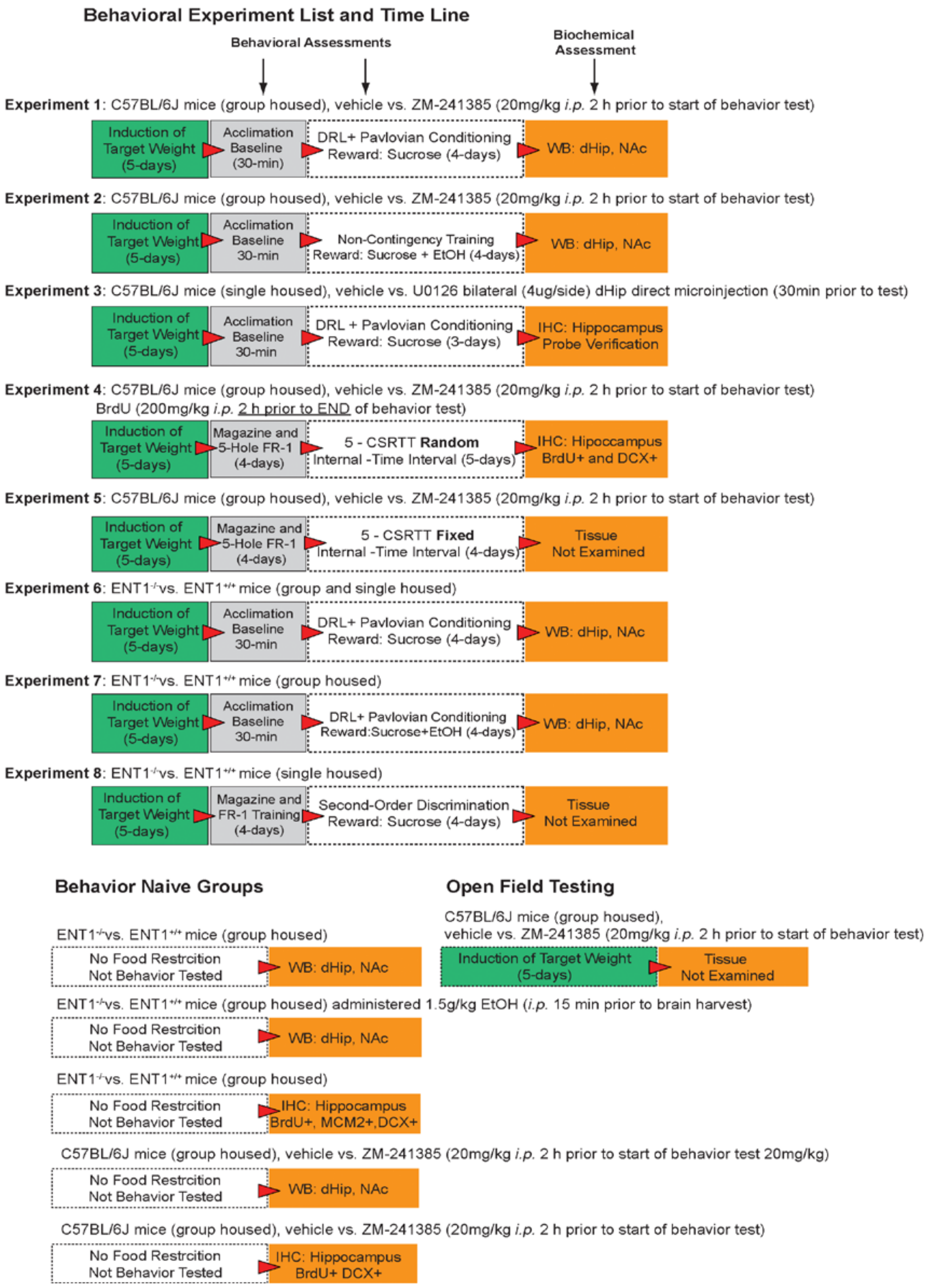

Supplementary Figure 1. Compiled schematic of experimental design, behavior testing and follow up biological assessments.

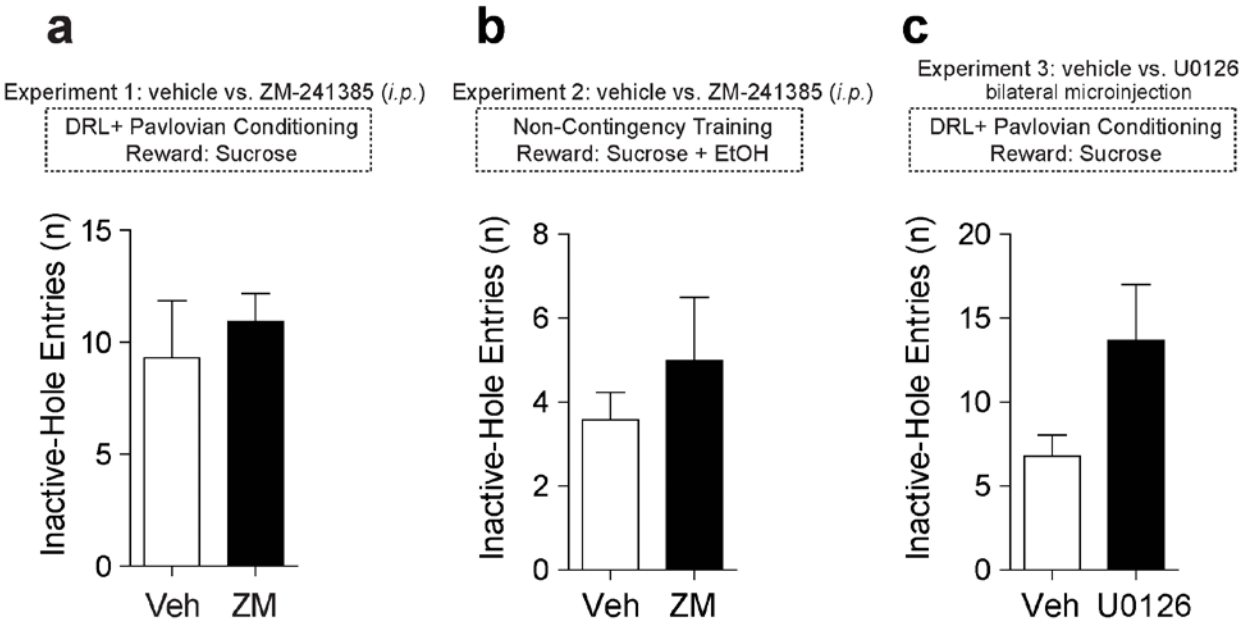

Supplementary Figure 2. Relative to vehicle, C57BL/6J mice administered (*i.p.*) ZM-241385 does not affect inactive hole entries during (**a**) DRL-conditioning (n = 5-6/treatment), (**b**) non-contingency training (n = 9-10/treatment). (**c**) There were no differences detected between the U0126 and vehicle treatment groups in general activity as measured by inactive-hole responses during conditioning (n = 3-4/treatment). Unpaired Student's *t*-test. All data are reported as mean ± SEM.

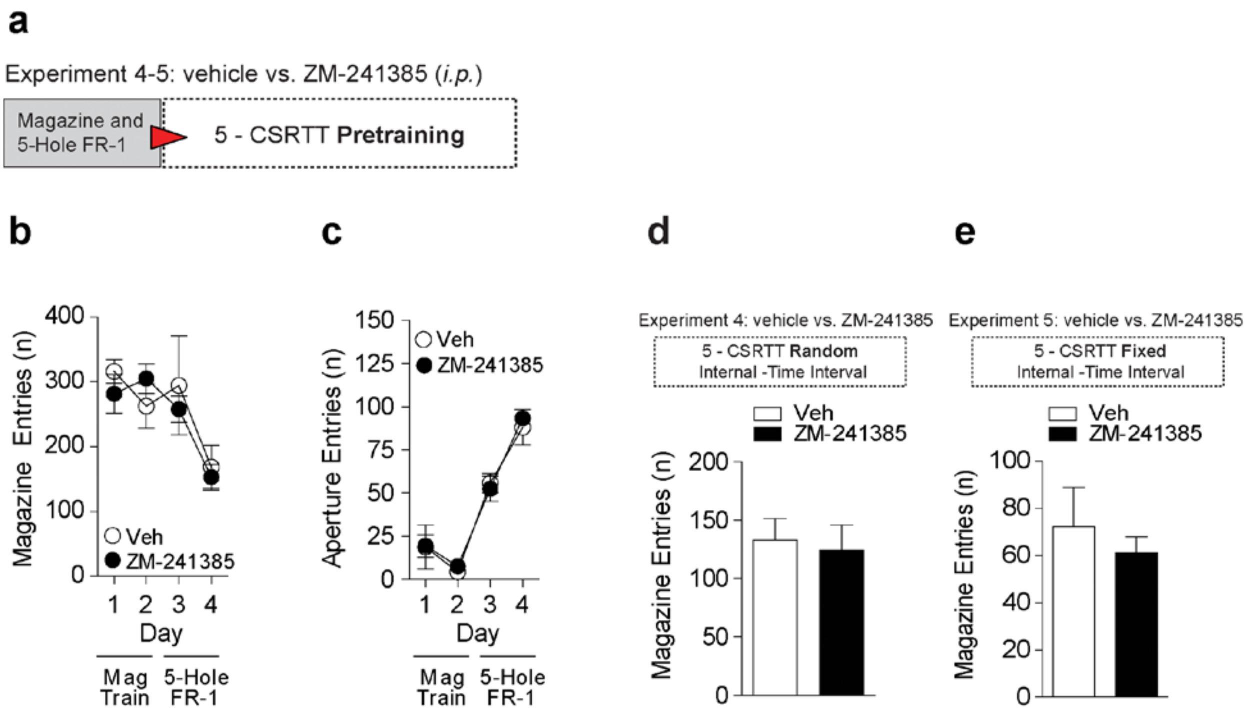

Supplementary Figure 3. (**a**) Schematic of experimental design for 5-choice serial reaction time task (5-CSRTT) pre-training. (**b**) Differences in magazine-entries measured during magazine training and 5-hole FR1 training prior were not detected in C57BL/6J mice designated to receive either ZM-241385 or vehicle (*i.p.*) during the 5CSRTT. (**c**) Similarly, aperture entries measured during magazine training and 5-hole FR1 training were not different between mice designated to receive either ZM-241385 or vehicle during 5CSRTT testing. (**d**) There were no differences detected in the number of magazine-entries during 5-CSRTT performance between the treatment groups when mice had to wait for unpredictable reinforcement-associated cues (random internal-time interval). (**e**) There were no differences between the treatment groups in the number of magazine-entries during 5-CSRTT performance when mice had to wait for predictable reinforcement-associated cues (fixed internal-time interval). **b**-**c**: n = 5-6/treatment designation, RM-two way ANOVA. **d**-**e**: n = 5-6/ treatment, unpaired two-tailed Student's *t*-test. All data are reported as mean ± SEM.

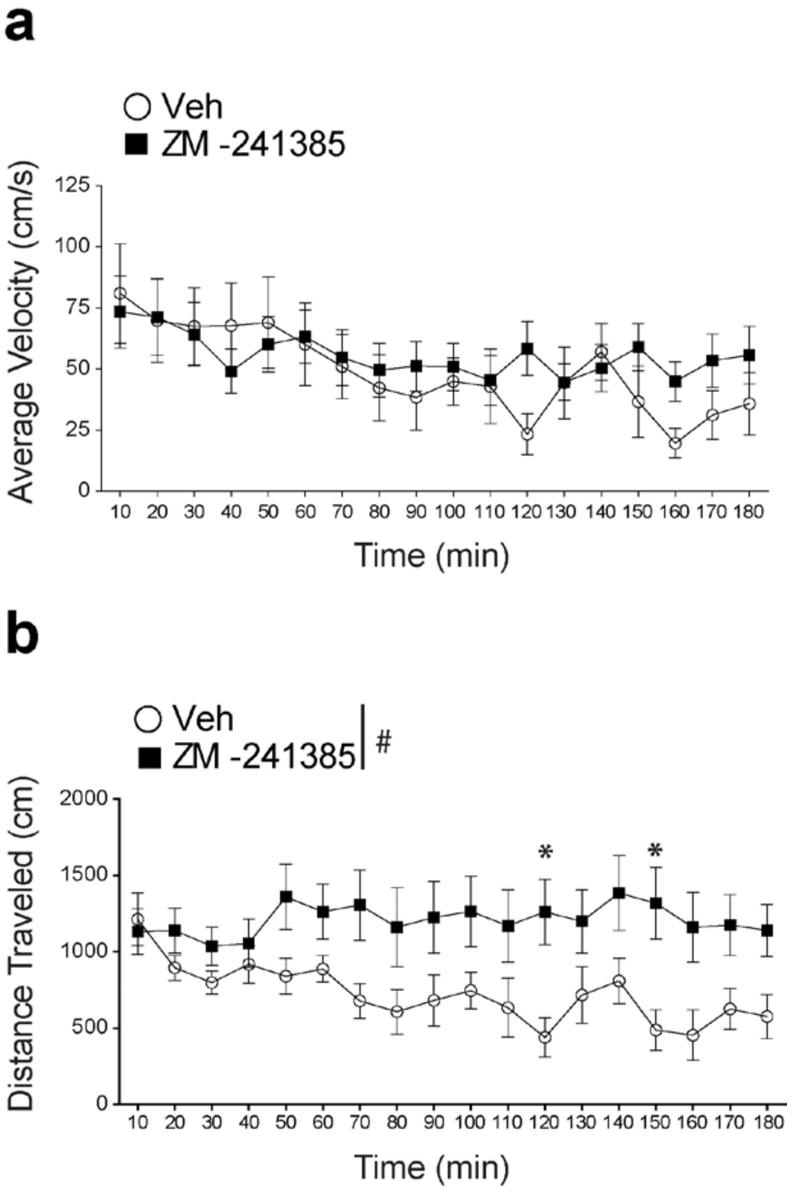

Supplementary Figure 4. (**a**) Open-field analysis was unable to detect differences in ambulatory velocity in mice treated with ZM-241385 or vehicle (*i.p.*). (**b**) Distance traveled was significantly maintained in C57BL/6J mice treated with ZM-241385 relative to vehicle. **a**-**b**: n = 9-10/treatment, RM two-way ANOVA. All data are reported as mean ± SEM.

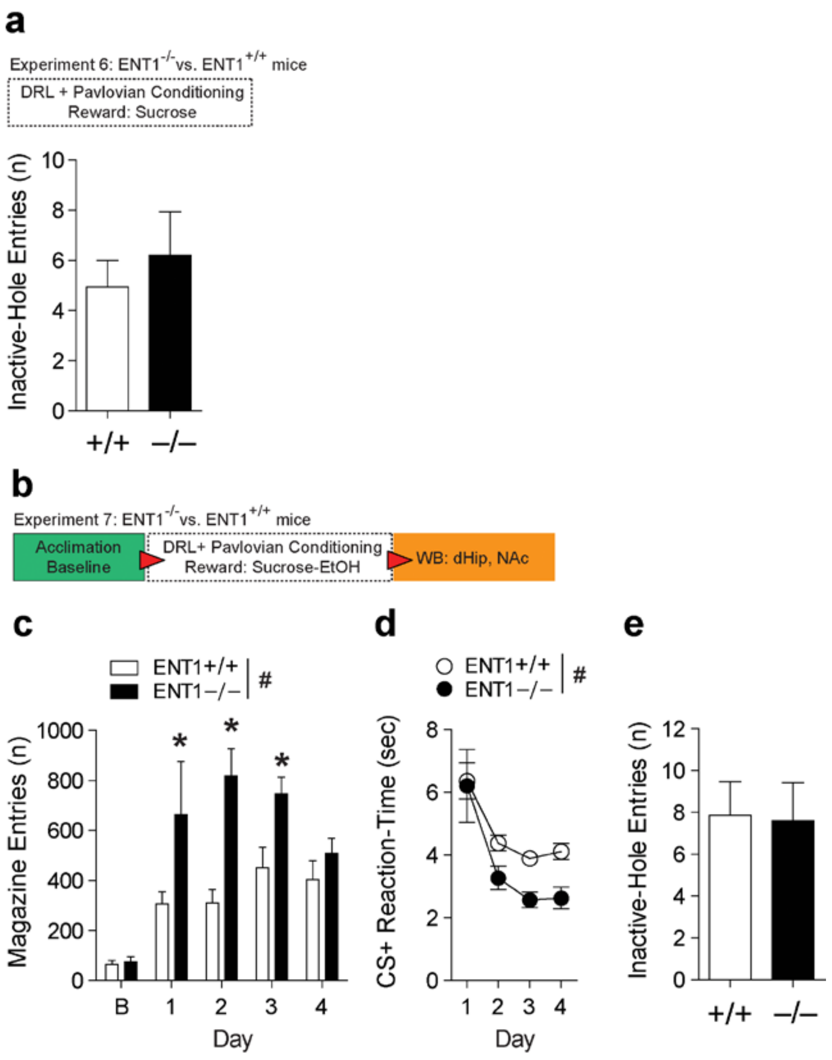

Supplementary Figure 5. (**a**) Analysis of general activity as measured by inactive-hole responses did not reveal differences between the genotypes during conditioning for sucrose reward (n = 13-15/genotype). (**b**) Schematic of experimental design for testing impulsivity during Pavlovian conditioning. (**c**) ENT1$^{-/-}$ mice display significantly higher magazine-entries for sucrose reward during conditioning. (**d**) In comparison to WT (+/+) mice, ENT1$^{-/-}$ mice display significantly faster reaction times following CS+ presentations to retrieve a sucrose-ethanol reward during conditioning. (**e**) Measurement of inactive-hole entries was not different between the genotypes during conditioning for sucrose-ethanol reward. All data are reported as mean ± SEM. RM two-way ANOVA, #$p < 0.05$ main effect of genotype; *$p < 0.05$ by Tukey's *post-hoc* multiple comparisons vs. WT mice. (**d**-**f**: n = 6-9/genotype).

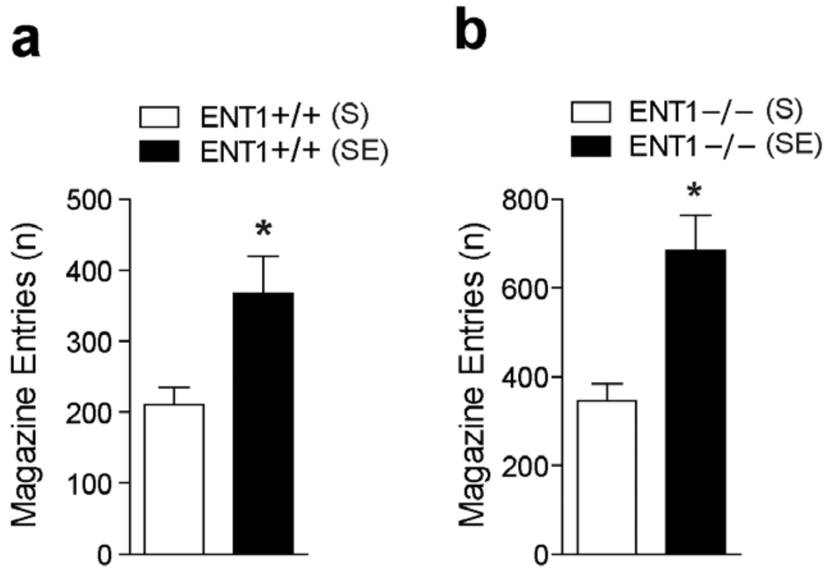

Supplementary Figure 6. Ethanol self-administration exacerbates goal-tracking impulsivity. (**a**) Relative to sucrose reward (n = 15), WT (+/+) mice display significantly higher impulsive magazine-entries for sucrose-ethanol reward during conditioning (n = 9). (**b**) Relative to sucrose reward (n = 13), ENT1$^{-/-}$ mice display significantly higher impulsive magazine-entries for sucrose-ethanol reward during conditioning. (n = 6). All data are reported as mean ± SEM. **p* < 0.05 by unpaired two-tailed *t*-test.

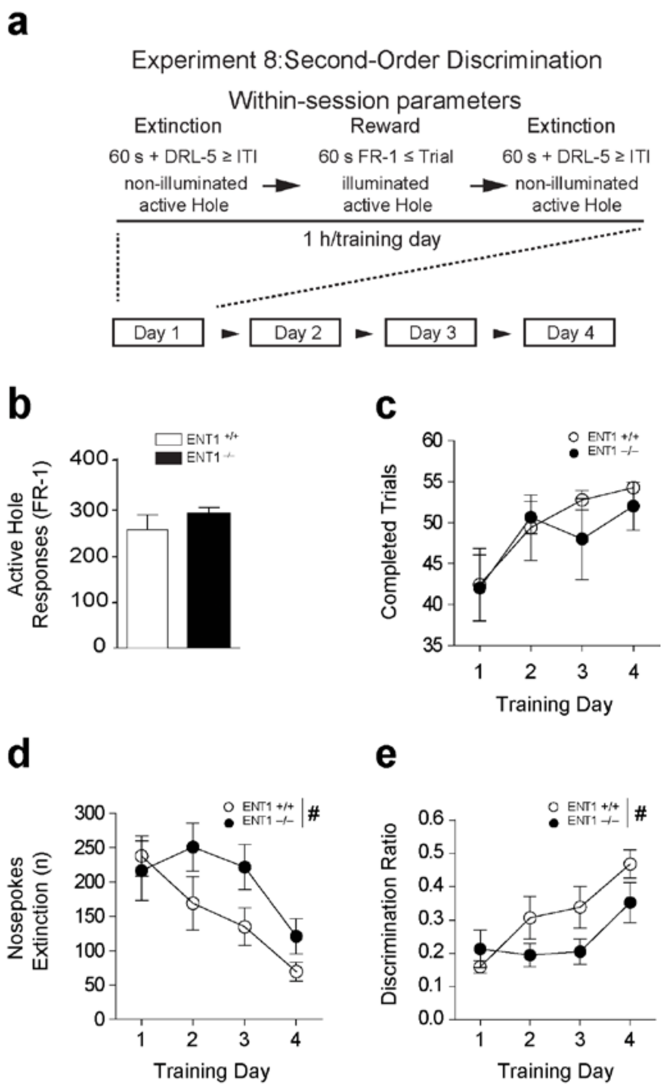

Supplementary Figure 7. (**a**) Schematic of the discrimination task. Operant second-order discrimination training was conducted daily for four days. Discrimination sessions were divided into inter-trial intervals (ITI ≥ 60 s; nosepokes on the non-illuminated active-hole were not reinforced, i.e. extinction), followed by onset of trials (active-hole illumination ≤ 60 s). A nosepoke into the non-illuminated active hole in the last 5 s of the ITI (DRL-5) delayed subsequent trial onset by 5 s. Correct responses (nosepokes during active hole illumination) were signaled by a 0.25 s tone, delivered reinforcement, turned off hole illumination, and began the next ITI. Response omission during a trial turned off illumination and started the next ITI. (**b**) There were no differences between WT (+/+) and ENT1$^{-/-}$ mice in active-hole responses for sucrose reward during FR1 pre-training. (**c**) During discrimination training, trial completions were not different between WT and ENT1$^{-/-}$ mice. (**d**) Relative to WT, ENT1$^{-/-}$ mice displayed significantly higher ITI nosepokes on the non-illuminated active-hole. (**e**) In comparison to WT, ENT1$^{-/-}$ mice show impaired discrimination performance as evidenced by a higher ratio of ITI active-hole nosepokes relative to correct nosepokes during trials. All data are expressed as mean ± SEM. Two-way ANOVA, #$p < 0.05$, main effect of genotype (**b**: n = 12-13/genotype; **c** - **e**: n = 8/genotype).

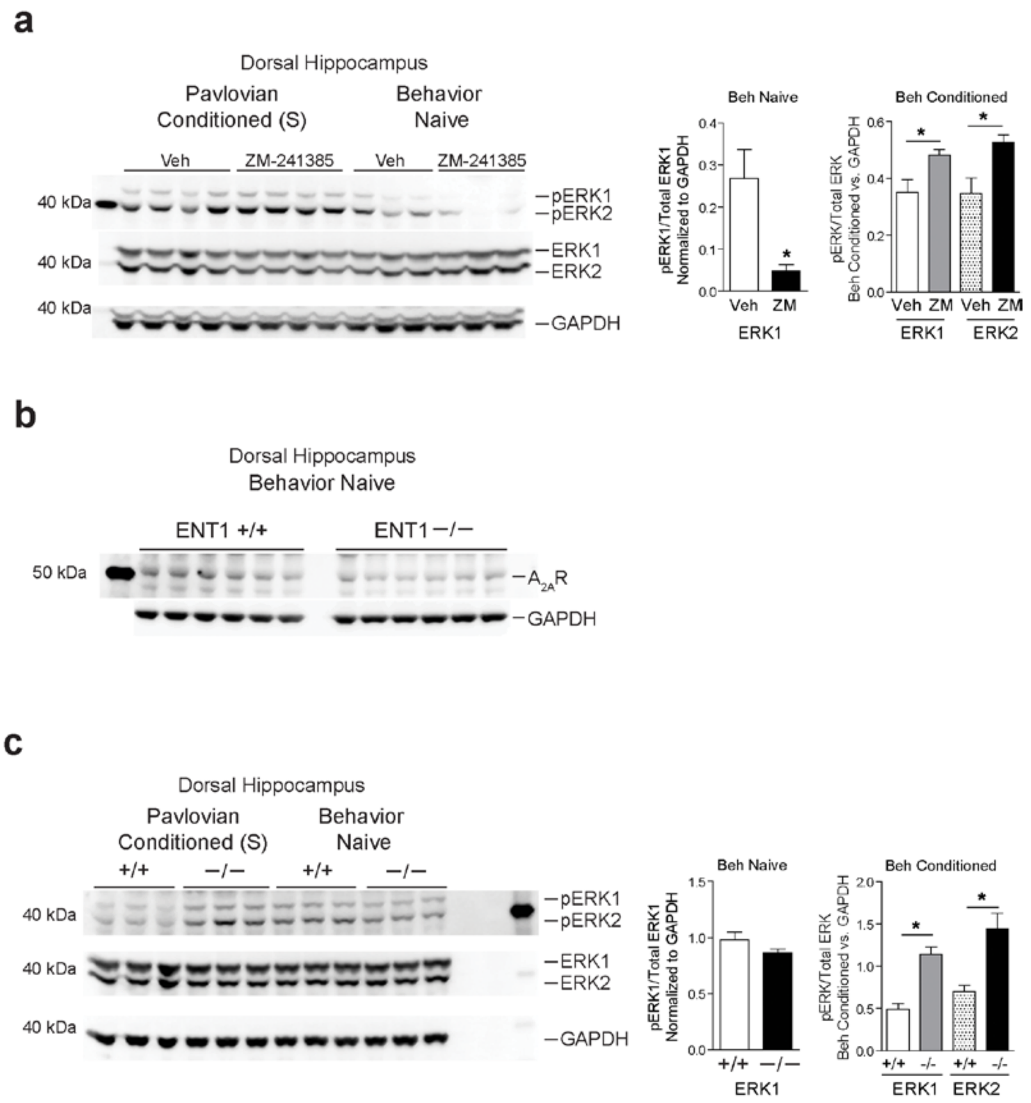

Supplementary Figure 8. Complete representative immunoblots of main text figures displaying protein expression in the dorsal hippocampus (dHip). (**a**) Relative to behavior (beh) naïve vehicle, ZM-241385 administration (*i.p.*) to beh naïve C57BL/6J decreases expression of phospho-ERK1 (n = 3/treatment) and phospho-ERK2 (see main text) in the dHip. In contrast, phospho-ERK1 and phospho-ERK2 were significantly higher in the dHip of mice treated with ZM-241385 which underwent beh conditioning vs. vehicle treated mice. *n* = 4/treatment. (**b**) Basal $A_{2A}R$ expression is decreased in the dHip of beh naïve $ENT1^{-/-}$ mice vs. WT (+/+) mice. *n* = 11/genotype. (**c**) Relative to beh naïve WT, beh naïve $ENT1^{-/-}$ mice display decreased expression of phospho-ERK1 (n = 3/treatment) and phospho-ERK2 (see main text) in the dHip. In contrast, phospho-ERK1 and phospho-ERK2 was significantly higher in the dHip of beh conditioned $ENT1^{-/-}$ mice vs. vehicle treated behavior mice. *n* = 3/genotype. All data are expressed as mean ± SEM. $*p < 0.05$ by unpaired two-tailed *t*-test.

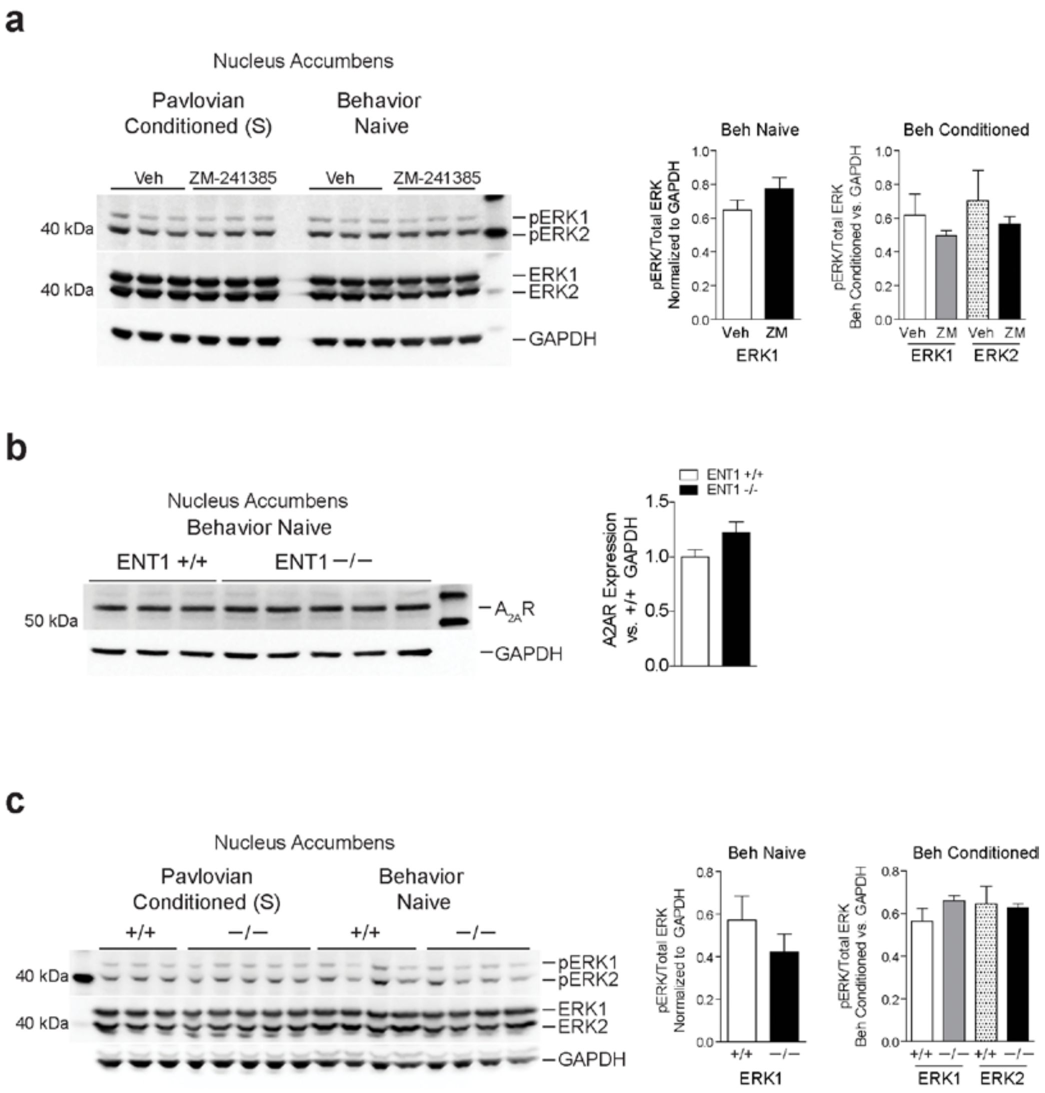

Supplementary Figure 9. Complete representative immunoblots of main text figures displaying protein expression in the nucleus accumbens (NAc). (**a**) There were no differences in phospho-ERK1 and phospho-ERK2 (see main text) expression in the dHip between behavior (beh) naïve vehicle and ZM-241385 treated (*i.p.*) C57BL/6J mice. There were also no differences detected in phospho-ERK1 and phospho-ERK2 in the dHip of mice treated with ZM-241385 or vehicle following beh conditioning. n = 3/treatment. (**b**) There were no differences in basal $A_{2A}R$ expression in the NAc of beh naïve $ENT1^{-/-}$ mice vs. WT (+/+) mice. n = 3-5/genotype. (**c**) There were no differences in phospho-ERK1 and phospho-ERK2 (see main text) expression in the NAc of behavior (beh) naïve $ENT1^{-/-}$ mice vs. WT mice (n = 4/genotype). Similarly, there were no differences detected in phospho-ERK1 and phospho-ERK2 expression in the NAc of mice treated with ZM-241385 or vehicle following beh conditioning. n = 3-5/genotype. All data are expressed as mean ± SEM. $*p < 0.05$ by unpaired two-tailed t-test.

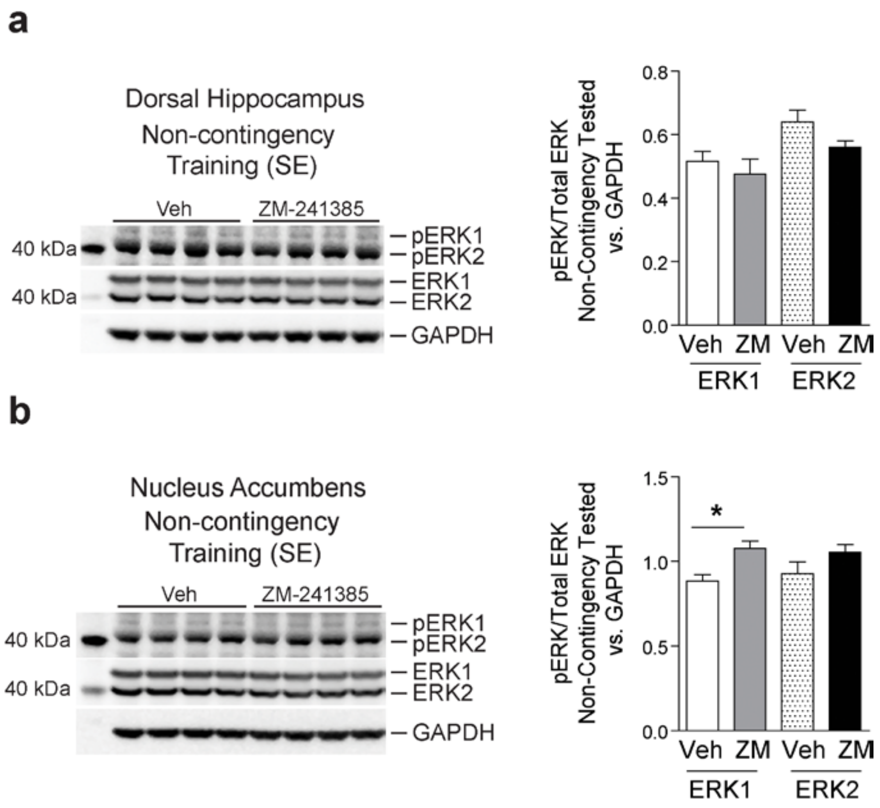

Supplementary Figure 10. Complete representative immunoblots displaying protein expression in the dorsal hippocampus (dHip) and nucleus accumbens (NAc) from C57BL/6J mice which underwent non-contingency testing.(**a**) There were no differences in phospho-ERK1 and phospho-ERK2 expression in the dHip between non-contingency trained vehicle and ZM-241385 treated (*i.p.*) mice n = 4/treatment. (**b**) Our analysis detected a significant increase in expression of phospho-ERK1 but not phospho-ERK2 in the NAc of mice that underwent non-contingency training and were administered ZM-241385 or vehicle. n = 4/treatment. All data are expressed as mean ± SEM. *$p < 0.05$ by unpaired two-tailed *t*-test.

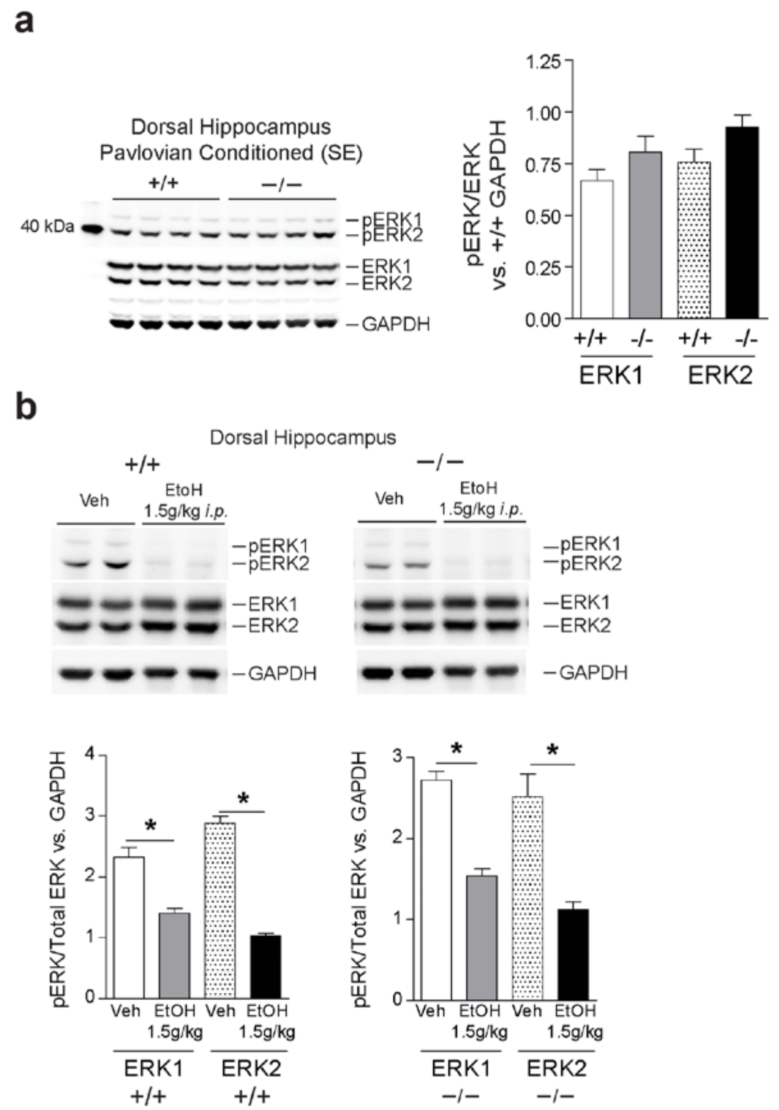

Supplementary Figure 11. Representative immunoblots showing the effects of ethanol on ERK1/2 phosphorylation in the dorsal hippocampus (dHip). (**a**) Complete immunoblot showing a lack of differences detected between ENT1$^{-/-}$ mice and WT mice in phospho-ERK1 and phospho-ERK2 expression in the dHip following DRL-conditioning for sucrose-ethanol (SE) reward. *n* = 4 mice/genotype. (**b**) Representative immunoblot showing a significant decrease of phospho-ERK1 and phospho-ERK2 expression in the dHip of WT (+/+) mice (left) treated (*i.p.*) with ethanol (1.5g/kg) vs. vehicle). *n* = 6/genotype and n = 3/treatment. One-way ANOVA, $F_{(3, 11)} = 62.59$, $p < 0.0001$,*$p < 0.01$ by Tukey's multiple comparisons. There was a significant decrease in phospho-ERK1 and phospho-ERK2 expression in the dHip of ENT1$^{-/-}$ mice (right) treated (*i.p.*) with ethanol (1.5g/kg) vs. vehicle. *n* = 6/genotype and n = 3/treatment. One-way ANOVA, $F_{(3, 11)} = 21.27$, $p < 0.001$,*$p < 0.01$ by Tukey's multiple comparisons. All data are expressed as mean ± SEM.

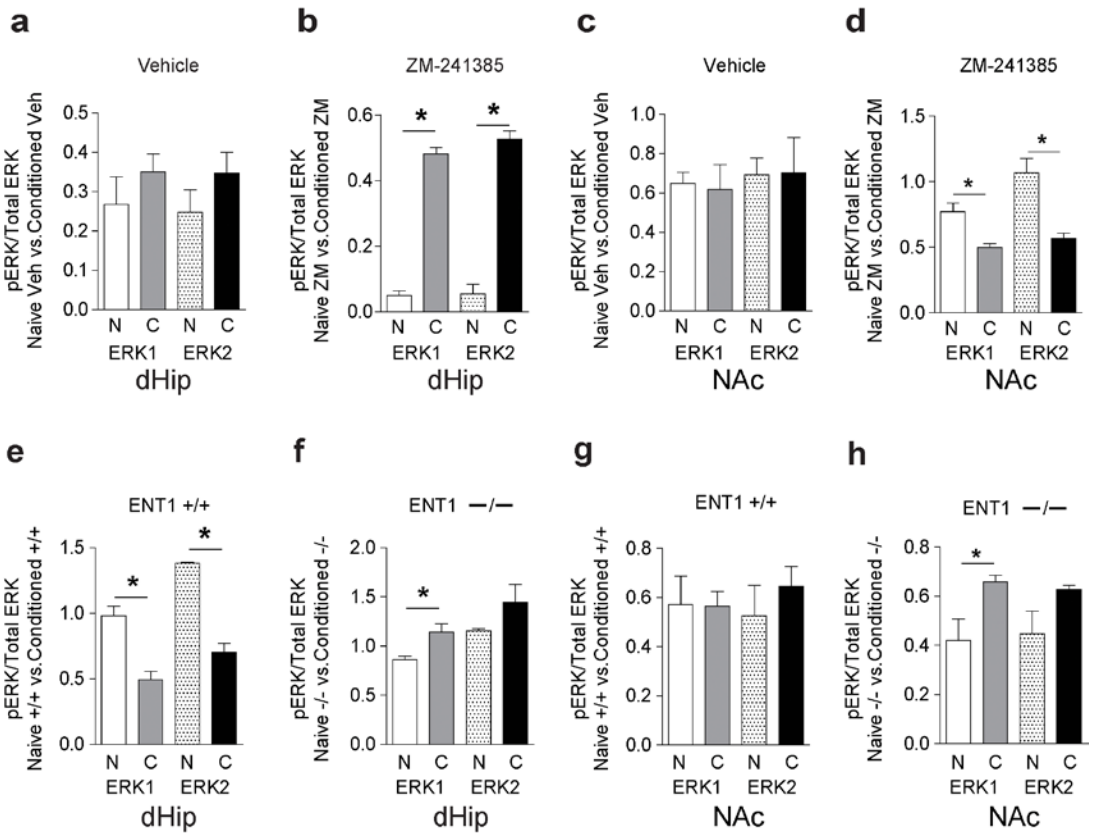

Supplementary Figure 12. Phospho-ERK activity in the dorsal hippocampus (dHip) and nucleus accumbens (NAc) as a result of DRL-conditioning. (**a**) There were no differences of phospho-ERK1 or phospho-ERK2 expression in the dHip between behavior naïve vehicle treated mice and DRL-conditioned vehicle treated mice. n = 3-4/behavioral condition. (**b**) There was a significant increase in expression of phospho-ERK1 and phospho-ERK2 in the dHip between behavior naïve ZM-241385 treated mice and DRL-conditioned ZM-241385 treated mice. n = 3-4/behavioral condition. (**c**) There were no differences in NAc expression of phospho-ERK1 or phospho-ERK2 between behavior naïve vehicle treated mice and DRL-conditioned vehicle treated mice. n = 3/behavioral condition. (**d**) There was a significant decrease in expression of phospho-ERK1 and phospho-ERK2 in the NAc between behavior naïve ZM-241385 treated mice and DRL-conditioned ZM-241385 treated mice. n = 3/behavioral condition. (**e**) There was a significant decrease in expression of phospho-ERK1 and phospho-ERK2 in the NAc between behavior naïve WT (+/+) mice and DRL-conditioned WT mice. n = 3/behavioral condition. (**f**) There was a significant increase in expression of phospho-ERK1 but not phospho-ERK2 in the dHip between behavior naïve ENT1$^{-/-}$ mice and DRL-conditioned ENT1$^{-/-}$ mice. n = 3/behavioral condition. (**g**) There were no differences in NAc expression of phospho-ERK1 or phospho-ERK2 between behavior naïve WT mice and DRL-conditioned WT mice. n = 3-4/behavioral condition. (**h**) There was a significant increase in expression of phospho-ERK1 but not phospho-ERK2 in the NAc between behavior naïve ENT1$^{-/-}$ mice and DRL-conditioned ENT1$^{-/-}$ mice. n = 4-5/behavioral condition. All data are expressed as mean ± SEM. *$p < 0.05$ by unpaired two-tailed *t*-test. N, behavior naïve; C, behavior conditioned.

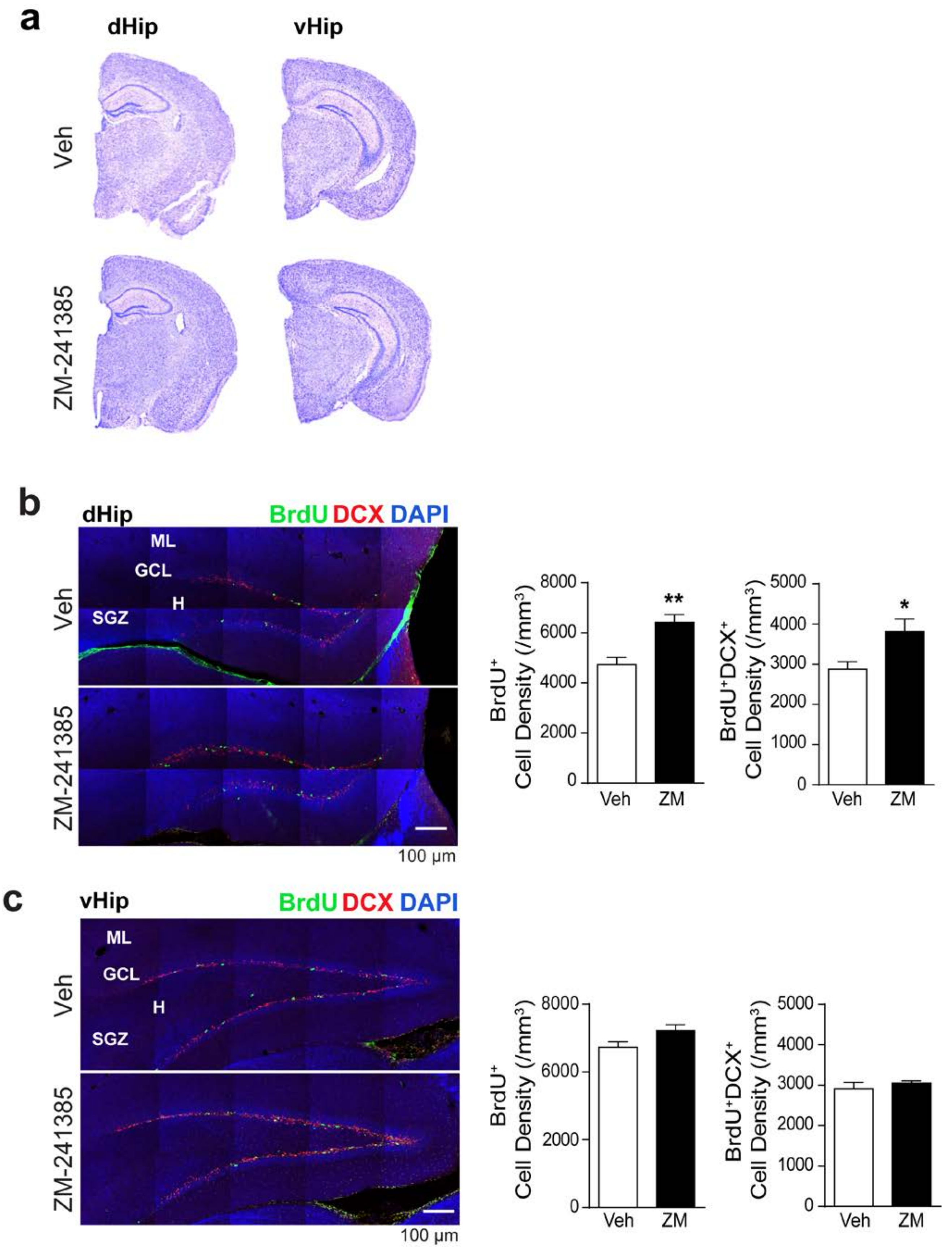
a
dHip
vHip
Veh
ZM-241385
b
dHip
BrdU DCX DAPI
ML
GCL
H
SGZ
Veh
ZM-241385
100 µm
BrdU⁺ Cell Density (/mm³)
BrdU⁺DCX⁺ Cell Density (/mm³)
8000
6000
4000
2000
0
5000
4000
3000
2000
1000
0
**
*
Veh
ZM
c
vHip
BrdU DCX DAPI
ML
GCL
H
SGZ
Veh
ZM-241385
100 µm
BrdU⁺ Cell Density (/mm³)
BrdU⁺DCX⁺ Cell Density (/mm³)
8000
6000
2000
0
5000
4000
3000
2000
1000
0
Veh
ZM

Supplementary Figure 13. Expression of markers for cell proliferation and neuroblast development in the dentate gyrus of the dorsal hippocampus (dHip) and ventral hippocampus (vHip) is differentially affected by $A_{2A}R$ inhibition mediated 5-CSRTT impulsivity. (**a**) Representative light microscopy images of the dHip and vHip. (**b**) Representative confocal microscopy images and stereological quantification analysis of $BrdU^+$ labeled cells and DCX^+ indicate significantly higher expression of $BrdU^+$ and $BrdU^+DCX^+$ co-labeling in the dHip of 5-CSRTT tested mice administered ZM-241385 relative to vehicle. DAPI (blue). Scale bars 100µm. n = 5-6 mice/treatment and n = 3 sections/dHip. $^{**}p < 0.01$ by unpaired two-tailed Student's *t*-test. (**c**) Representative confocal microscopy images and stereological quantification analysis of $BrdU^+$ and $BrdU^+DCX^+$ co-labeling in the vHip of mice did not detect differences between mice administered ZM-241385 relative to vehicle. Scale bars 100µm. n = 5-6 mice/treatment and n = 3 sections/vHip. $^{*}p < 0.05$ by unpaired two-tailed Student's *t*-test. $BrdU^+$ (green), DCX^+ (red), DAPI (blue). Subgranular zone (SGZ), Dentate gyrus (DG) of the hippocampus. GCL, Granular cell layer; H, Hilus; ML, Molecular layer. All data are reported as mean ± SEM.

SUPPLEMENTARY MATERIALS AND METHODS

Subjects

ENT1$^{-/-}$ and wild type (WT) mice were generated in house, while C57BL/6J mice were ordered from Jackson Laboratories (Bar Harbor, ME USA). All animals were generated and cared for under a 12 hr/12 hr light-dark cycle with lights on at 06:00 and off at 18:00, as previously described [1, 2]. All mice utilized in this study were age-matched 3-to-5 month male mice. A list of all the experimental groups and procedures is depicted in Supplementary Figure 1. All experimental procedures were approved by the Mayo Clinic Institutional Animal Care and Use Committee in accordance with NIH guidelines.

Behavior Naïve Mice. ENT1$^{-/-}$, WT, and C57BL/6J mice that were naïve to behavior testing and were acutely administered ethanol, or were treated with ZM-241385 (5-days), were group-housed with littermates and maintained in ventilated racks with *ad libitum* food and water until ready for brain extraction for subsequent western blot and immunofluorescence analysis as described below (Supplementary Figure 1).

Behavior Tested Mice. Groups tested for Pavlovian conditioning, Fixed Ratio-1 (FR1) training, second-order discrimination, non-contingency training and open-field (Supplementary Figure 1), ENT1$^{-/-}$, WT, and C57BL/6J mice were individually housed and group housed (see Supplementary Figure 1 for details) and food restricted to reach approximately 85% of their *ad libitum* feeding weight (target weight). For 5-CSRTT pre-training (magazine training, 5-hole FR1 and 5-CSRTT) and 5-CSRTT testing, C57BL/6J mice were group housed and food restricted to reach target weight (Supplementary

Figure 1). All subjects were weighed daily and allowed 30 m to acclimate to behavior procedure rooms prior to testing. Immediately following each behavior test, mice were returned to their home cage and fed a daily ration of rodent chow in order to maintain target weight. The behavioral measurements and programming parameters for each behavior test were computer controlled and recorded by Med-PC IV software using Med-State notation (Med-Associates, Inc.). All programs utilized and modified for the experiments in this study can be found at http://www.mednr.com/index.htm as part of the MEDstate Notation repository.

Drugs

For all experiments involving ZM-241385 (20 mg/kg; *i.p.,* Tocris Bioscience, Bristol, UK), the specific $A_{2A}R$ antagonist was dissolved in 15% DMSO, 15% Cremophor, and in 0.9% saline (Sigma-Aldrich, St. Louis MO, USA), at a dose of 0.1ml/10g of body weight. To investigate the effects of acute ethanol administration on protein expression in the dorsal hippocampus (dHip), male ENT1$^{-/-}$and WT mice were administered an injection of ethanol at a concentration of 1.5 g/kg, *i.p.*; 20% (v/v), dissolved in isotonic saline followed by brain extraction 15 min post injection. To inhibit ERK1/2 in the dHip, the specific MEK inhibitor U0126 (1,4-diamino-2,3-dicyano-1,4-bis (o-aminophenylmercapto) butadiene monoethanolate) was dissolved in 50% DMSO and 0.9% saline (Sigma-Aldrich).

DRL mediated Pavlovian conditioning

We used Pavlovian and operant conditioning to measure impulsive goal tracking and premature sign-tracking impulsivity [3, 4]. During baseline and Pavlovian conditioning, mice were allowed to freely explore to the operant chamber (MED-307A-B2, Med-Associates, Inc.) with the cubicle fan (CF) and the house light (HL) turned on. Total session time (ST), right and left nosepokes (inactive-holes), and magazine head-entries (magazine-entries) were recorded (ENV-302HD head entry detector, Med-Associates, Inc.). During baseline (30 min), inactive-hole entries (measure of general activity) and magazine entries had no programmed consequences. Following baseline, mice underwent Pavlovian conditioning [5, 6] where they were presented with a random series of tone cues (65dB and 0.25 s in duration; ENV-323HAM, 4500 Hz Sonalert, Med-Associates, Inc.). Each randomly presented tone (CS+) was paired with delivery (PHM-100 syringe pump, Med-Associates, Inc.) of a reward (10µl/reward) and recorded by the computer, thus establishing a tone-reward contingency [7]. Acquisition of Pavlovian conditioning was determined by measuring the latency between a CS+ and the next-immediate CS+ evoked magazine entry (CS+ reaction time). The resulting latencies were summed and then divided by the number of CS+ to determine an average reaction time following CS+ presentations.

To induce impulsive response behavior, we programmed a DRL schedule into the Pavlovian conditioning task [8]. This schedule utilizes a random time interval between CS+ presentations (the shortest interval being 2 s and the longest being 30 s), so that on average a reward was delivered every 15 s. Any interruption of the magazine entry detector in the absence of a CS+ restarts the time interval before presentation of the next CS+. Session termination was signaled by the HL and CF shutting off, and

determined by reaching a pre-defined number of CS+ presentations (as described below). Mice were then immediately placed into their home cage, except on the final testing day where mice were sacrificed immediately following conditioning to isolate the dHip and NAc for western blot analysis.

Pavlovian Conditioning and Non-Contingency Experiments.

Experiment 1. Group housed C57BL/6J mice (n = 5-6/treatment) received 20% sucrose (maximum of 50 CS+ at 10 µl/reward) to examine the effects of the specific $A_{2A}R$ antagonist ZM-241385 or vehicle, on impulsivity during Pavlovian conditioning. Mice were injected with ZM-241385 two hours prior to conditioning, as described previously [2, 9].

Experiment 2. Non-Contingency Training. To ascertain the effect of $A_{2A}R$ antagonism in the absence of Pavlovian conditioning, group housed C57BL/6J mice (n = 10/treatment) were injected with ZM-241385 or vehicle (as described for *Experiment 1*) and randomly presented with a series of neutral stimulus tones (maximum of 40 CS-), with the shortest interval being 5 s and the longest being 90 s, so that on average a CS- was presented every 30 s [7, 10], Delivery of non-contingent rewards (20% sucrose + 10% ethanol; maximum of 40) were followed by a series of random time intervals, with the shortest interval being 2 s and the longest being 60 s, so that on average a reward was delivered every 15 s. For this experiment, a DRL schedule was not implemented. Data parameters acquired were identical to *Experiment 1*. Immediately following the final training session, mice were sacrificed and the dHip and NAc were harvested for western blot analysis as described above.

Experiment 3. Group housed C57BL/6J mice (n = 3-4/treatment) underwent baseline and one training day of Pavlovian conditioning (sucrose reward, maximum of 60 CS+ at 10 µl/reward) before *ad libitum* feeding in order to prepare for cannulation surgery. Following cannulation surgery and recovery, mice were single housed and directly infused with U0126 or vehicle 30 min prior to DRL-conditioning to test the effects of dampened ERK1/2 phosphorylation on goal-tracking impulsivity. Vehicle and U0126 were bilaterally infused (4µg/µL/side) at a rate of 0.5µL/min for a total of 2 min.

Operant Conditioning Experiments

5-CSRTT Testing

5-CSRTT pre-training. Magazine training and 5-hole FR1 training required for 5-CSRTT acquisition was conducted in five-aperture operant conditioning chambers (MED-NP5M-D1, Med Associates, Inc.). Except for *Habituation*, session duration, aperture responses, and reward magazine entries were recorded for each training stage. *Habituation.* To encourage aperture exploration, 50 µl of reward solution was pipetted into each aperture (L-100 *Pipette-Lite;* Mettler-Toledo International, Inc.) and mice were allowed to explore the chambers for 15 min/day for 2 days. Magazine entries and nosepokes were not recorded. *Magazine Training.* Training (30 m sessions daily/3 days) consisted of random illumination of any one of the 5 apertures paired with reward deliveries (20 µl) signaled by illumination of the reward magazine. Reward was available for 5 s after which the reward magazine light and aperture light turned off and

the dipper retracted. Between reward deliveries, a random computer controlled ITI consisted of a minimum of 2 s and a maximum of 60 s. After a maximum of 50 reward deliveries, the HL turned off and the session terminated. *5-Hole FR1 Training.* Next, we established an association between the 5-hole apertures and reinforcement delivery. During training sessions, all five nosepoke holes were illuminated and a nosepoke response at any aperture resulted in reinforcement, which required a magazine head entry. Reinforcement was available for a maximum of 5 s, after which the magazine light extinguished and the dipper retracted. Sessions were terminated after 30 reinforcements on day 1, and 50 reinforcements on days 2-3.

5-CSRTT. Following FR1 training, we adapted the 5-CSRTT in order to assess impulsive response behavior [11]. To further assess the effects of $A_{2A}R$ inhibition on sign-tracking impulsivity, we utilized 5-aperture operant conditioning chambers (Med-Associates, Inc.) to test the 5-CSRTT [11]. Briefly, this task consists of time out intervals (TOI, 5 s) that are followed by trials. Trials are composed of an internal trial-interval (INT-TI) which is followed by random illumination of any one of the 5 apertures within the chamber. A nosepoke into an illuminated aperture or within 5 s following extinction of the aperture light (limited hold) is recorded as a correct trial and results in reinforcement (similar as described for 5-hole FR1). Aperture nosepokes during an INT-TI restarts the INT-TI and is recorded as an impulsive premature response. Similarly, aperture nosepokes during TOIs resets the TOI, delaying trial onset and serves as a measure of impulsivity. A nosepoke on any hole except the illuminated aperture turns off the light, initiates the next trial and is recorded as an incorrect

response. Failure to nosepoke into an illuminated or non-illuminated aperture during a trial is recorded as an omission, and initiates the next trial. Sessions were terminated after 30 min or 50 trials had elapsed.

Experiment 4. Group housed C57BL/6J mice were treated with either vehicle (n = 5) or ZM-241385 (n = 6) daily for 5 days. Aperture illumination was fixed at 2 s. For reinforcement, mice had to wait for random (ranged from a minimum of 2 s to a maximum of 60 s) internal time-interval durations (rINT-TI) to expire before nosepoking, thus making reinforcement-associated aperture illuminations unpredictable. TOIs remained constant at 5 s.

Experiment 5. In this experiment, we sought to examine the effects of $A_{2A}R$ antagonism in conjunction with a longer aperture illumination time and a fixed INT-TI. In this experiment, Group housed C57BL/6J mice were treated with either vehicle (n = 5) or ZM-241385 (n = 6) as described for *Experiment 4*. Aperture illumination was gradually shortened from a duration of 10 s (training days 1-2) to 2 s (training days 3-4). For reinforcement, mice had to wait for fixed (5 s) internal time-interval durations (fINT-TI) to expire before nosepoking an illuminated aperture, thus making reinforcement-associated aperture illuminations predictable. TOIs remained constant at 5s.

Experiment 6. WT and $ENT1^{-/-}$ mice (n = 13-15/genotype) received 20% sucrose (maximum of 50 CS+ at 10 µl/reward) during DRL-conditioning. Mice in this experiment

were both single housed and group housed. Behavioral programming and data acquisition parameters for *Experiment 6* were identical as described for *Experiment 1*.

Experiment 7. In order to investigate the effects of a more hedonic reward on impulsivity during DRL-conditioning, group housed WT and ENT1$^{-/-}$ mice (n = 6-9/genotype) of 20% sucrose and 10% ethanol (maximum of 60 CS+ at 10 µl/reward). Behavioral programming and data acquisition parameters for *Experiment 7* were identical as described for *Experiment 1*.

Second-Order Discrimination.

FR1 Training. To test the effects of ENT1 deletion on acquisition of stimulus discrimination (i.e. responses when nosepoke hole was illuminated vs. extinction of responses when the nosepoke hole was not illuminated), single housed WT and ENT1$^{-/-}$ mice (n = 12-13/genotype) first underwent magazine training, and shaping by successive approximation [12] to establish FR1 nosepoke responses for sucrose reward. For FR1 training and discrimination testing (see below), correct active-hole nosepoke responses were paired with a 0.25 s tone, while any responses on the inactive-hole (non-illuminated left nosepoke hole) were recorded but had no programmed consequences.

Discrimination Testing. Following FR1 training, WT and ENT1$^{-/-}$ mice (n = 8/genotype) underwent second-order discrimination [5, 13]. As depicted in Supplementary Figure 6a, this task consisted of mixed, fixed trials (fixed interval ≤ 60 s, signaled by active-hole

illumination), followed by inter-trial intervals (ITI $\geq$ 60 s duration). To assess impulsivity during ITIs, a DRL-5 schedule was implemented so that nosepokes into the non-illuminated active-hole during the last 5 s of the ITI delayed trial onset by 5 s. An FR1 response during a trial turned off the illuminated active-hole, delivered reinforcement and started the next ITI. Response omissions during trials turned off active-hole illumination and commenced the next ITI. Using the equation below, we examined responses on the non-illuminated active-hole to determine the extent of extinguished responses during the ITI [14]. Sessions were terminated after 1 hr or after 60 trials.

Equation 1.

$$\frac{Completed\ FR1\ Trials}{(\boldsymbol{C}ompleted\ FR1\ Trials + Responses\ during\ ITI)}$$

Open-field Testing. To determine the effects of $A_{2A}R$ antagonism on open field activity, group housed C57BL/6J mice were injected (*i.p.*) with ZM-241385 or vehicle (n = 9-10/treatment) and assessed for average velocity (cm/s) and distance traveled (cm) as previously described [15].

Cannulation and Microinjection Procedure.

For *Experiment 3*, we utilized a double-guided cannulae injection system (C315G(2)-G11/SP; PlasticsOne, Roanoke VA, USA) which included injectors extending 1mm beyond the guide cannulae, a dummy cannula (internal 33ga; guide 26ga) and a cap. Surgeries targeting the dHip (AP: -2.1mm from Bregma; Lateral: ±1.8mm, and DV:-

1.6mm below the dural surface [16] were performed as previously described [15]. Following cannulation surgery, mice were allowed to recover for 6-7 days before starting food restriction weight as described above. To ensure the health of our subjects during conditioning following cannulation surgeries, we used their pre-surgery *ad libitum* feeding weight to calculate their *post-surgery* target weight. Approximately 30 min prior to Pavlovian conditioning, mice were briefly exposed (20-30 s) to isofluorane anesthesia and either vehicle or U0126 (4µg/µL/side) was bilaterally infused with a glass syringe and microinjection pump (CMA Microdialysis, Sweden) at a rate of 0.5µL/min for a total of 2 min. Injections were allowed to diffuse for an additional 2 min before removal of injectors. During the infusions, mice were secured by gently holding the tail and were allowed to ambulate as they recovered from isofluorane anesthesia.

Tissue Preparation

Tissue Extraction for Western Blot and Immunofluorescence. All mice were subjected to rapid CO_2 inhalation to induce unconsciousness, followed by decapitation and subsequent harvesting of brain for isolation of the dHip and NAc from both hemispheres under a surgical microscope. The extracted tissue was snap-frozen on dry ice and stored at -80°C until it was processed for SDS-PAGE (Criterion, Bio-Rad Laboratories, Hercules CA, USA) and western blotting as described below. For immunofluorescence, mice receiving ZM-241385 or vehicle were injected with BrdU (200mg/kg body weight, *i.p.*) at a dose of 0.1ml/10g of body weight 1h post $A_{2A}R$ antagonist or vehicle treatment, and incubation of BrdU was allowed for 2 h as mice were 1) left in the home cage for behavior naïve mice or 2) as the mice performed 5-CSRTT testing [17]. Mice treated with

ZM-241385 or vehicle were injected *(i.p.)* with a ketamine (100mg/kg) and xylazine (7mg/kg) mixture at a dose of 0.1ml/10g of body weight to induce unconsciousness prior to perfusion. After perfusion, brains were harvested and placed in 4% paraformaldehyde (1x PBS) for 24 h. Subsequently, fixed brains were cryoprotected by incubation in 30% sucrose, until ready for cryostat slicing.

Western Blot.

We examined basal protein expression of the $A_{2A}R$ (n = 11/genotype) and ERK1/2 (n = 3/genotype) in the dHip of naïve $ENT1^{-/-}$ and WT mice. We also examined basal protein expression of the $A_{2A}R$ (n = 3-5/genotype) as well as phosphorylated and total ERK1 and ERK2 (n = 4/genotype) in the NAc of naïve $ENT1^{-/-}$ and WT mice. Likewise, we examined protein expression in the dHip following ZM-241385 in C57BL/6J mice that were naïve to behavior (*n* = 3/treatment). We also explored changes in expression of phosphorylated and total ERK1 and ERK2 in the dHip following Pavlovian conditioning in $ENT1^{-/-}$ (*n* = 3), WT (*n* = 3), and C57BL/6J mice treated with vehicle (*n* = 3) or with ZM-241385 (*n* = 3) (Supplementary Figure 1). In addition, we investigated changes in expression of phosphorylated and total ERK1 and ERK2 in the NAc following Pavlovian conditioning in $ENT1^{-/-}$ (*n* = 5), WT (*n* = 3), and C57BL/6J mice treated with vehicle (*n* = 3) or with ZM-241385 (*n* = 3) (Supplementary Figure 1). Furthermore, we examined changes in expression of phosphorylated and total ERK1 and ERK2 in the dHip (n = 4/treatment) and the NAc (n = 4/treatment) following non-contingency training in C57BL/6J mice treated with vehicle or with ZM-241385 (Supplementary Figure 1).

The dHip and NAc from each mouse were homogenized in a Storm 24 magnetic Bullet Blender for 4 min at a speed setting of 4 (Next Advance Inc., Averill Park NY, USA), with 0.5 mm zirconium oxide beads in combination with 50-70 µl of Cell-lytic MT mammalian tissue extraction reagent (Sigma-Aldrich) containing 50 mM Tris buffer (pH 7.4), 2 mM EDTA, 5 mM EGTA, and 0.1% SDS. The homogenization buffer also contained Complete (Roche) protease inhibitor cocktail and phosphatase inhibitor cocktails type II and III (Sigma-Aldrich). Homogenates were then centrifuged at 16,400 rpm (4°C) for 15 min and supernatants were collected. Protein concentration from each replicate supernatant was quantified using the Bradford protein assay (Bio-Rad).

Brain region replicates from each animal were loaded at 30 µg and separated on a 4-12% Nu-Page Bis-Tris gel in MOPS buffer (Invitrogen, Carlsbad, CA) at 130 V for 2 hr, followed by transfer to a PVDF membrane (Invitrogen) at 30 V for 1 hr. Samples were then immunoblotted overnight at 4°C (5% BSA in 1x TBST) with primary antibodies specific for the $A_{2A}R$ (1:500, 45 kDa; Millipore, Billerica MA, USA), ERK1/2 (1:500, 42 and 44 kDa; Cell Signaling Technology Inc., Danvers MA, USA), phospho-ERK1/2 (1:500, 42 and 44 kDa; Cell Signaling), and GAPDH (1:1000, 38 kDa; Millipore). Following three 10 min washes in 1x TBST, respective anti-rabbit and anti-mouse secondary antibodies (1:1000, Millipore) were incubated in 1x TBST with immunoblots for 1 hr at room temperature. Blots were incubated for 5 min with the Super-Signal West Dura Chemiluminescent Substrate (Thermo Scientific, Rockford, IL), developed on a Kodak Image Station 4000R scanner (New Haven, CT). Each immunoblot was exposed to detect chemoluminescence for a minimum of 5 s and not exceeding 30 s, in

order to avoid potential exposure saturation. Band optical density quantification was performed using NIH ImageJ software.

Immunofluorescence.

We examined expression of markers indicative of cell and neuroblast proliferation (BrdU$^+$,MCM2$^+$ and DCX$^+$) from the whole hippocampal DG (5 sections/brain, 200μm apart from anterior to posterior) in age matched (6-week old) behavior naïve WT and ENT1$^{-/-}$ mice (n = 4-5/genotype). Similarly, we examined the process of neuroblast proliferation (BrdU$^+$DCX$^+$) in 5-CSRTT tested mice administered with ZM-241385 or vehicle (n = 5-6/treatment). From these behavior tested mice we investigated the whole hippocampus (5 sections/brain, 200μm apart from anterior to posterior) and separately the dHip (3 sections/region, 200μm apart from anterior to posterior) and ventral hippocampus (vHip; 3 sections/region, 200μm apart from anterior to posterior) to assess region specific differences. Sample volumes were determined from these sections and cell density was multiplied by the total volume to yield the absolute cell numbers [17, 18].Coronal brain sections (40μm thick) from each mouse brain were processed to label proliferating cells with primary antibodies for BrdU$^+$ (1:250), MCM2$^+$ (BM28;1:500), and neuroblasts in the stage of maturation where doublecortin (DCX$^+$;1:500) is expressed. Appropriate secondary antibodies (Cy2, Cy3 and Cy5) were used to detect primary antibodies [17, 19]. Images were acquired on a LSM 780 confocal system (Zeiss) with X20 and X40 objectives using a multi-tile configuration. Stereological quantification of BrdU$^+$, BrdU$^+$DCX$^+$, and BrdU$^+$MCM2$^+$ cells within the subgranular zone (SGZ) and granule cell layer of the DG were carried out using Zen Blue edition (Zeiss) as

previously described [17, 18]. To localize cell nuclei, slices were stained with 4', 6-diaminodino-2-phenylindole (DAPI, 1:5000, Santa Cruz).

Data and Statistical analysis

Pavlovian conditioning and non-contingency testing: For CS+ and CS- reaction times, as well as magazine-entries, we conducted repeated measures two-way ANOVA (RM two-way ANOVA) or two-way ANOVA where appropriate. Inactive-hole responses from each subject were averaged for all training days analyzed with an unpaired two-tailed Student's *t*-test. For comparison of magazine-entries between sucrose and sucrose-ethanol reward for each genotype, magazine-entries were averaged for all training days analyzed with an unpaired two-tailed Student's *t*-test. D*iscrimination*: For completed trials, ITI nosepokes and discrimination ratios we used two-way ANOVA. *5-CSRTT*: For magazine-entries, aperture entries, FR1 rewards, and premature nosepokes during 5-CSRTT pre-training and 5-CSRTT testing, we used RM two-way ANOVA. For TOI responses, magazine-entries, % correct, % incorrect and % omission trials during 5-CSRTT testing, each subject's performance was averaged for all training days, and compared with unpaired two-tailed Student's *t*-test. *Open field*: Velocity and distance traveled were analyzed with RM two-way ANOVA.

For western blot analysis, each lane sample was an individual brain region biological replicate from WT and ENT1$^{-/-}$ mice or vehicle and ZM-241385. Each biological replicate was normalized to its respective GAPDH protein expression. Unless otherwise specified, we utilized an unpaired Student's *t*-test or a one-way ANOVA

followed by Tukey's multiple comparisons for western blot analysis. Images are representative of 1-3 Western blotting experiments. For all $BrdU^+$, $BrdU^+MCM2^+$, and $BrdU^+DCX^+$ statistical analyses, cell numbers derived from 3-5 coronal sections were averaged for each brain and we utilized an unpaired Student's *t*-test for comparisons. Statistical significances reported for all ANOVA results were followed with Tukey's multiple comparisons where appropriate (Sigma Plot 12.0, Systat Software San Jose CA, USA). Results were considered statistically significant when $p < 0.05$ and are presented as mean and ±SEM (GraphPad Prism, La Jolla CA, USA).

References

1. Choi DS, Cascini MG, Mailliard W, Young H, Paredes P, McMahon T *et al.* The type 1 equilibrative nucleoside transporter regulates ethanol intoxication and preference. *Nat Neurosci* 2004; **7**(8)**:** 855-861.

2. Nam HW, Hinton DJ, Kang NY, Kim T, Lee MR, Oliveros A *et al.* Adenosine transporter ENT1 regulates the acquisition of goal-directed behavior and ethanol drinking through A2A receptor in the dorsomedial striatum. *J Neurosci* 2013; **33**(10)**:** 4329-4338.

3. Flagel SB, Watson SJ, Akil H, Robinson TE. Individual differences in the attribution of incentive salience to a reward-related cue: influence on cocaine sensitization. *Behav Brain Res* 2008; **186**(1)**:** 48-56.

4. Winstanley CA. The utility of rat models of impulsivity in developing pharmacotherapies for impulse control disorders. *Br J Pharmacol* 2011; **164**(4)**:** 1301-1321.

5. Keller FS, Schoenfeld WN. *Principles of psychology; a systematic text in the science of behavior*. Appleton-Century-Crofts: New York,, 1950, xv, 431 p.pp.

6. Mead AN, Stephens DN. Selective disruption of stimulus-reward learning in glutamate receptor gria1 knock-out mice. *J Neurosci* 2003; **23**(3)**:** 1041-1048.

7. Rescorla RA. Pavlovian conditioning and its proper control procedures. *Psychol Rev* 1967; **74**(1)**:** 71-80.

8. Chance P. *Learning and behavior*. Seventh edition. edn. Thomson and Wadsworth, 2013, xviii, 446 pagespp.

9. Stella SL, Jr., Bryson EJ, Cadetti L, Thoreson WB. Endogenous adenosine reduces glutamatergic output from rods through activation of A2-like adenosine receptors. *J Neurophysiol* 2003; **90**(1)**:** 165-174.

10. Rescorla RA. Probability of shock in the presence and absence of CS in fear conditioning. *J Comp Physiol Psychol* 1968; **66**(1)**:** 1-5.

11. Carli M, Robbins TW, Evenden JL, Everitt BJ. Effects of lesions to ascending noradrenergic neurones on performance of a 5-choice serial reaction task in rats; implications for theories of dorsal noradrenergic bundle function based on selective attention and arousal. *Behav Brain Res* 1983; **9**(3)**:** 361-380.

12. Boules M, Iversen I, Oliveros A, Shaw A, Williams K, Robinson J *et al.* The neurotensin receptor agonist NT69L suppresses sucrose-reinforced operant behavior in the rat. *Brain Res* 2007; **1127**(1)**:** 90-98.

13. Di Ciano P, Everitt BJ. Neuropsychopharmacology of drug seeking: Insights from studies with second-order schedules of drug reinforcement. *Eur J Pharmacol* 2005; **526**(1-3)**:** 186-198.

14. Featherstone RE, McDonald RJ. Dorsal striatum and stimulus-response learning: lesions of the dorsolateral, but not dorsomedial, striatum impair acquisition of a stimulus-response-based instrumental discrimination task, while sparing conditioned place preference learning. *Neuroscience* 2004; **124**(1)**:** 23-31.

15. Vadnie CA, Hinton DJ, Choi S, Choi Y, Ruby CL, Oliveros A *et al.* Activation of neurotensin receptor type 1 attenuates locomotor activity. *Neuropharmacology* 2014; **85:** 482-492.

16. Franklin KaP, G. *The Mouse Brain in Stereotaxic Coordinates.* 3 edn. Elsevier Academic: San Diego, 2007.

17. Jang MH, Bonaguidi MA, Kitabatake Y, Sun J, Song J, Kang E *et al.* Secreted frizzled-related protein 3 regulates activity-dependent adult hippocampal neurogenesis. *Cell Stem Cell* 2013; **12**(2)**:** 215-223.

18. Kempermann G, Kuhn HG, Gage FH. More hippocampal neurons in adult mice living in an enriched environment. *Nature* 1997; **386**(6624)**:** 493-495.

19. Bonaguidi MA, Wheeler MA, Shapiro JS, Stadel RP, Sun GJ, Ming GL *et al.* In vivo clonal analysis reveals self-renewing and multipotent adult neural stem cell characteristics. *Cell* 2011; **145**(7)**:** 1142-1155.
